# Nuclear Control of Mitochondrial Homeostasis and Venetoclax Efficacy in AML via COX4I1

**DOI:** 10.1002/advs.202404620

**Published:** 2024-12-23

**Authors:** Leisi Zhang, Honghai Zhang, Ting‐Yu Wang, Mingli Li, Anthony K.N. Chan, Hyunjun Kang, Lai C. Foong, Qiao Liu, Sheela Pangeni Pokharel, Nicole M. Mattson, Priyanka Singh, Zeinab Elsayed, Benjamin Kuang, Xueer Wang, Steven T. Rosen, Jianjun Chen, Lu Yang, Tsui‐Fen Chou, Rui Su, Chun‐Wei David Chen

**Affiliations:** ^1^ Department of Systems Biology Beckman Research Institute City of Hope 1500 E Duarte Rd Duarte CA 91010 USA; ^2^ National Clinical Research Center for Hematologic Diseases Jiangsu Institute of Hematology The First Affiliated Hospital of Soochow University 296 Shizi St Suzhou Jiangsu 215005 China; ^3^ Proteome Exploration Laboratory California Institute of Technology 1200 E California Blvd Pasadena CA 91125 USA; ^4^ Division of Epigenetic and Transcriptional Engineering Beckman Research Institute City of Hope 1500 E Duarte Rd Duarte CA 91010 USA; ^5^ Department of Hematologic Malignancies Translational Science Beckman Research Institute City of Hope 1500 E Duarte Rd Duarte CA 91010; ^6^ City of Hope Comprehensive Cancer Center 1500 E Duarte Rd Duarte CA 91010 USA

**Keywords:** chlorpromazine, COX4I1, leukemia, mitochondria, venetoclax

## Abstract

Cell signaling pathways are enriched for biological processes crucial for cellular communication, response to external stimuli, and metabolism. Here, a cell signaling‐focused CRISPR screen identified cytochrome c oxidase subunit 4 isoform 1 (COX4I1) as a novel vulnerability in acute myeloid leukemia (AML). Depletion of COX4I1 hindered leukemia cell proliferation and impacted in vivo AML progression. Mechanistically, loss of COX4I1 induced mitochondrial stress and ferroptosis, disrupting mitochondrial ultrastructure and oxidative phosphorylation. CRISPR gene tiling scans, coupled with mitochondrial proteomics, dissected critical regions within COX4I1 essential for leukemia cell survival, providing detailed insights into the mitochondrial Complex IV assembly network. Furthermore, COX4I1 depletion or pharmacological inhibition of Complex IV (using chlorpromazine) synergized with venetoclax, providing a promising avenue for improved leukemia therapy. This study highlights COX4I1, a nuclear encoded mitochondrial protein, as a critical mitochondrial checkpoint, offering insights into its functional significance and potential clinical implications in AML.

## Graphical abstract

Using a cell signaling‐focused CRISPR screen, this study revealed COX4I1 as a new vulnerability in acute myeloid leukemia (AML) via maintaining mitochondrial ultrastructure and oxidative phosphorylation. CRISPR gene tiling scans and mitochondrial proteomics identified key COX4I1 regions crucial for Complex IV assembly. Targeting COX4I1 in combination with venetoclax shows promise for enhancing leukemia therapy.

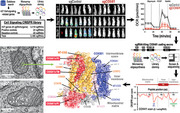

## 1 Introduction

In hematopoietic malignancies, acute myeloid leukemia (AML) stands as one of the most aggressive conditions in adults. More than 60% of AML patients exhibit mutations in signal transduction pathways, including those resulting in abnormal activation of signaling pathways associated with prognosis.^[^ ^1^ ^]^ Over the past five decades, cytotoxic chemotherapy coupled with bone marrow transplant has been the cornerstone of AML treatment. With an in‐depth comprehension of the etiology and pathobiology of AML, there has been a surge in therapies targeting distinct molecular pathways of AML.^[^ ^2^ ^]^ Nonetheless, the 5‐year survival rate of AML patients in the USA has remained at ≈30 percent (NIH Surveillance, Epidemiology, and End Results Program; https://seer.cancer.gov/statfacts/html/amyl.html). Therefore, broadening the treatment spectrum for patients and marking a significant shift in the AML management approach is urgently needed.

Malignant cell proliferation is characterized by the aberration of normal intracellular signaling, driven by mutations or aberrant external signaling. Cell signaling encompasses a fundamental array of biological mechanisms crucial for regulating cellular processes such as proliferation, metabolism, apoptosis, gene expression, and interaction with the extracellular environment. The orchestration of cell signaling involves intricately regulated pathways governing the expression and activation of signaling transducers and receptors, thereby connecting different cellular compartments to coordinate diverse cellular behaviors. Within eukaryotic cells, mitochondria serve as pivotal platforms for initiating, transducing, and receiving various signaling processes. This intracellular organelle central to cellular bioenergetics, plays a multifaceted role in cell growth, energy metabolism, programmed cell death (such as apoptosis and ferroptosis), and malignancy.^[^ ^3^ ^]^ Mitochondria are primarily responsible for adenosine triphosphate (ATP) synthesis, a vital molecule for cellular energy metabolism. ATP is predominantly generated within mitochondria via oxidative phosphorylation, a process where electrons generated by the citric acid cycle are transferred along the mitochondrial respiratory complexes.^[^ ^4^ ^]^ Additionally, mitochondria are integral to other essential cellular functions such as fatty acid synthesis, maintenance of iron homeostasis, modulation of growth factor signaling, responses to hypoxic stress, and modulation of immunity.^[^ ^5^ ^]^ Dysregulation of mitochondria is frequently implicated in various human diseases including cancers.^[^ ^6^ ^]^ Given the critical role of mitochondrion in both normal cellular function and disease pathology, a comprehensive understanding of its functions and regulatory mechanisms holds significant promise for future therapeutic advancements.

In this study, we employed an unbiased CRISPR/Cas9 library screen targeting major cell signaling pathway components, aiming to identify essential factors in leukemia. Our investigation pinpointed cytochrome c oxidase subunit 4 isoform 1 (COX4I1), a nuclear encoded mitochondrial protein, as crucial for leukemia survival. Leveraging genetic, transcriptomic, and proteomic analyses, we delineated the critical role of COX4I1 in maintaining mitochondrial ultrastructure and oxidative phosphorylation. Utilizing the CRISPR gene tiling approach combined with a high‐resolution structural genetic analysis,^[^ ^7^ ^]^ we elucidated specific regions of COX4I1 involved in various stages of mitochondrial Complex IV assembly. Our findings underscore the significance of COX4I1 in mitochondrial homeostasis, shedding light on potential avenues for mitochondria‐targeted therapies in AML.

## 2 Results

### 2.1 CRISPR Library Screen Focuses on Cell Signaling Pathways Unveiled COX4I1 as a Novel Vulnerability in Leukemia

**Figure 1:**
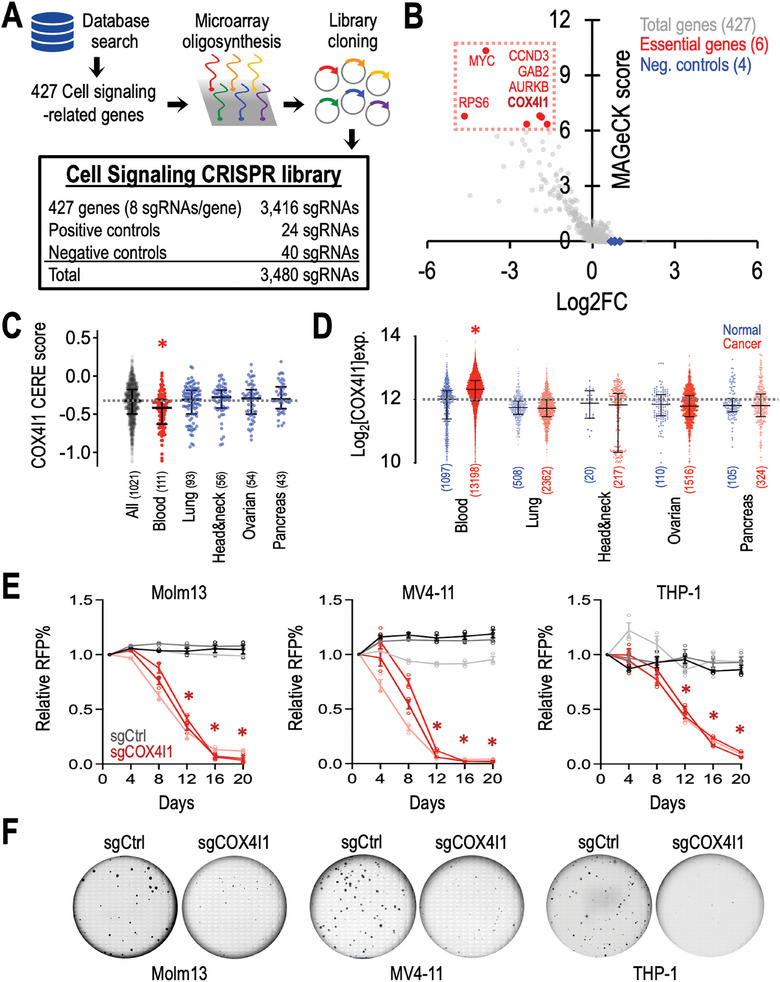
Identification of the essential role of COX4I1 in leukemia cells through cell signaling CRISPR screens. A) Schematic representation of the design and cloning process of the cell signaling CRISPR library. B) Volcano plot illustrating the log2 fold change of sgRNA abundance over 28 days of screen culture (x‐axis; log2FC) and the significance (y‐axis; MAGeCK score) of each gene in the cell signaling CRISPR screen of Molm13‐Cas9^+^ cells. Essential genes are highlighted in red, negative controls in blue, and the total library in gray. C) Dot plots showing the CRISPR gene dependency (CERE) score of COX4I1 across 1021 human cancer cell lines tested in the DepMap consortium (BROAD Institute). D) Expression profile of COX4I1 in normal and cancer samples, sourced from the GENT2 database (http://gent2.appex.kr/gent2/). E) Growth competition assay of Cas9‐expressing Molm13 (left), MV4‐11 (middle), and THP‐1 (right) cells transduced with RFP‐labeled sgCtrl (gray lines; *n* = 3 independent sgRNA sequences) and sgCOX4I1 (red lines; *n* = 3 independent sgRNA sequences). F) Representative images depicting leukemic blast colonies derived from Cas9‐expressing Molm13 (left), MV4‐11 (middle), and THP‐1 (right) cells transduced with sgCtrl and sgCOX4I1. Data are presented as (C and D) median ± interquartile range and (E) mean ± SEM. ^*^ *p* < 0.01 by two‐sided Student's *t*‐test.

To unravel critical components of cell signaling pathways essential for leukemia cell proliferation, we conducted an analysis of Gene Ontology (GO) pathways, encompassing “Intracellular signaling transduction,” “Cell communication,” “Protein phosphorylation,” “Cell surface receptor signaling pathway,” “Cellular response to chemical stimulus,” “Regulation of programmed cell death,” “Cellular response to an organic substance,” and “Phosphorus metabolic process”.^[^ ^8^ ^]^ This analysis yielded a list of 427 cell signaling‐related genes (SourceData S1, Supporting Information). Subsequently, we designed a focused CRISPR library targeting these genes, incorporating eight sgRNAs per gene (**Figure** **1**A; totaling 3416 sgRNAs). Additionally, we included a panel of 40 negative control sgRNAs (targeting non‐human genes such as Luc, LacZ, Ren, Rosa26, and scrambled sequences) and 24 positive control sgRNAs (targeting cancer essential genes such as PCNA, RPA3, CDK, etc.; SourceData S2 and Figure S1A,B, Supporting Information). Lentiviral transduction facilitated the delivery of this library into Cas9‐expressing Molm13 human leukemia cells, and the frequency of each integrated sgRNA construct was monitored on day 0 and day 28 using high‐throughput sequencing (SourceData S3, Supporting Information). Employing the MAGeCK algorithm,^[^ ^9^ ^]^ this focused CRISPR screen unveiled COX4I1 (encoding Cytochrome c oxidase subunit 4 isoform 1) as a top essential gene in leukemia cells, alongside five previously reported leukemia essential genes (Figure 1B, red; MYC, RPS6, CCND3, GAB2, and AURKB).

Further analysis of the cancer cell line CERE score, a computational method estimating gene‐dependency levels from CRISPR‐Cas9 essentiality screens,^[^ ^10^ ^]^ across 102 cell lines (Data source: https://depmap.org/portal/; BROAD Institute) (Figure 1C), revealed a significantly stronger survival dependency on COX4I1 in human blood malignancies (red; 111 cell lines) compared to other cancer cell types. Clinically, we observed selective overexpression of COX4I1 in patients with blood cancers (Figure 1D; GENT2 database),^[^ ^11^ ^]^ indicating a potential involvement of COX4I1 in hematopoietic cancers. To validate the screen results, we transduced Cas9‐expressing leukemia cells (including Molm13, MV4‐11, and THP‐1) with sgRNAs targeting COX4I1 (sgCOX4I1) and non‐essential sequences (sgCtrl). Utilizing a flow cytometric growth competition assay,^[^ ^7^, ^12^ ^]^ we demonstrated that leukemia cells transduced with sgCOX4I1 were outcompeted by cells transduced with sgCtrl (Figure 1E). CRISPR depletion of COX4I1 also resulted in reduced colony‐forming capacity of the leukemia cells (Figure 1F), highlighting the requirement of COX4I1 in leukemia cell proliferation.

**Figure 2:**
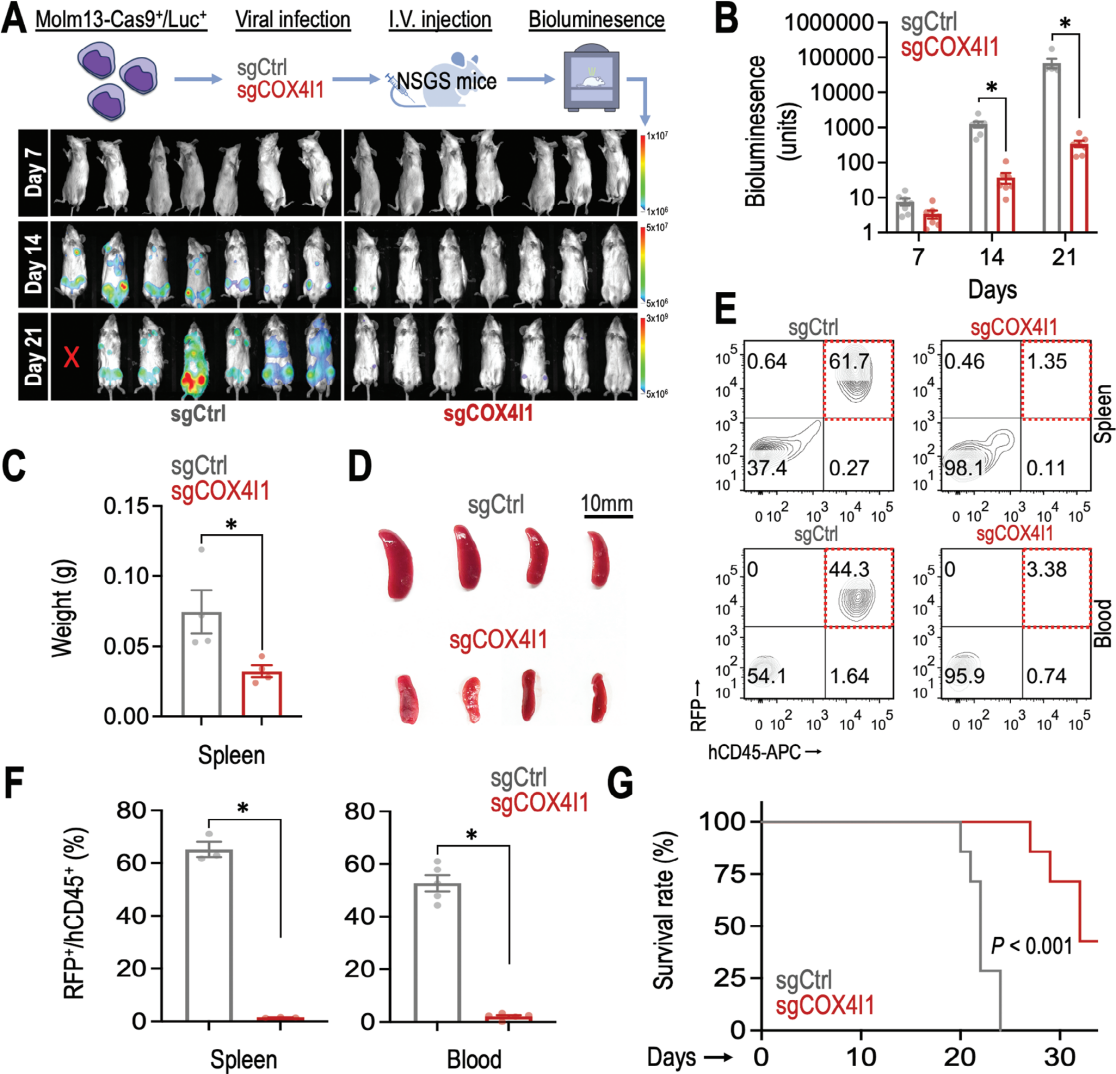
COX4I1 is indispensable for in vivo AML progression. A) Schematic representation of a “human‐in‐mouse” leukemia xenograft model and in vivo bioluminescent images using NSGS mice injected with Cas9/luciferase‐expressing (Cas9^+^/Luc^+^) human Molm13 AML cells transduced with sgCtrl and sgCOX4I1 (*n* = 7 mice per group). B) Quantitative analysis of bioluminescent signals from NSGS recipient mice transplanted with Molm13‐ Cas9^+^/Luc^+^ AML cells transduced with sgCtrl and sgCOX4I1 (*n* = 7 mice per group). C,D) Impact of COX4I1 depletion on (C) spleen weight and (D) spleen size (*n* = 4 mice per group). E,F) Representative flow cytometric profiles of RFP and human CD45 (hCD45) expression (E) and the percentage of RFP^+^/hCD45^+^ human AML cells in the spleen (*n* = 3) and peripheral blood (*n* = 5) of mice receiving Molm13‐Cas9^+^/Luc^+^ AML cells transduced with sgCtrl and sgCOX4I1 (F). G) Kaplan‐Meier survival curve of NSGS recipient mice transplanted with Molm13‐Cas9^+^/Luc^+^ AML cells transduced with sgCtrl and sgCOX4I1 (*n* = 7 mice per group). Data are presented as mean ± SEM. ^*^ *p* < 0.01 by two‐sided Student's *t*‐test.

To assess the impact of targeting COX4I1 on the maintenance of human leukemia, we transduced a Molm13‐Cas9^+^/Luc^+^ human acute myeloid leukemia (AML) model^[^ ^12^, ^13^ ^]^ with sgCtrl and sgCOX4I1. Subsequently, we transplanted these human AML cells into immunodeficient NSG‐SGM3 (NSGS) recipient mice and monitored leukemia progression using bioluminescence imaging (**Figure** **2**A). This “human‐in‐mouse” xenograft leukemia model revealed a significant reduction in leukemia burden upon COX4I1 depletion (Figure 2A,B, as detected by non‐invasive bioluminescence in vivo imaging). Consistent with this finding, depletion of COX4I1 inhibited splenomegaly (Figure 2C,D) and reduced engraftment of RFP^+^/hCD45^+^ human AML cells in the spleen and peripheral blood of recipient mice (Figure 2E,F). Importantly, CRISPR depletion of COX4I1 delayed leukemia onset in recipient mice (Figure 2G), providing proof‐of‐concept evidence of targeting COX4I1 in vivo to disrupt the progression of human AML.

### 2.2 Loss of COX4I1 Induces Mitochondrial Stress in Leukemia

**Figure 3:**
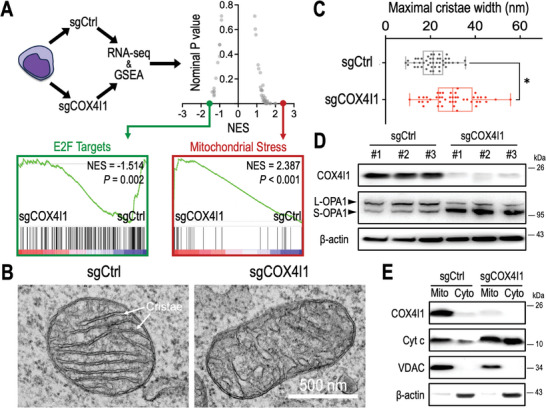
COX4I1 is crucial for maintaining mitochondrial homeostasis. A) RNA‐seq and Gene Set Enrichment Analysis (GSEA) revealing alterations in the expression of “E2F Targets” and “Mitochondrial Stress” gene sets in sgCtrl versus sgCOX4I1‐transduced Molm13‐Cas9^+^ cells. Each dot represents a Hallmark gene set from the GSEA Molecular Signature Database. NES indicates normalized enrichment score. B) Representative images from transmission electron microscopy depicting mitochondrial ultrastructure and C) measurement of maximal cristae width observed in Molm13‐Cas9^+^ cells transduced with sgCtrl and sgCOX4I1. D) Western blot analysis of COX4I1, OPA1, and β‐actin in Molm13‐Cas9+ cells transduced with sgCtrl (*n* = 3 independent sgRNA sequences) and sgCOX4I1 (*n* = 3 independent sgRNA sequences). E) Distribution of COX4I1, cytochrome c (cyt c), voltage‐dependent anion channel (VDAC; a mitochondrial marker protein), and β‐actin (a cytoplasmic marker protein) between the mitochondrial (mito) and cytoplasmic (cyto) fractions of Molm13‐Cas9^+^ cells transduced with sgCtrl and sgCOX4I1. Data are presented as (C) box‐and‐whisker plots illustrating median (center line), first and third quartiles (box), and data range (whiskers), and G and H) bar graphs showing mean ± SEM. ^*^ *p* < 0.01 by two‐sided Student's *t*‐test.

To delineate the consequences of COX4I1 depletion, we conducted RNA‐seq and gene set enrichment analysis (GSEA)^[^ ^14^ ^]^ in Molm13‐Cas9 cells transduced with sgCtrl versus sgCOX4I1. Our analysis revealed that the “E2F_Pathway” is significantly depleted among GSEA hallmark gene sets upon COX4I1 knockout, indicating a pivotal role of COX4I1 in supporting cellular proliferation (**Figure** **3**A, green). Conversely, genes associated with the mitochondrial stress response^[^ ^15^ ^]^ were notably enriched in COX4I1‐deficient Molm13 cells (Figure 3A; red). Subsequently, transmission electron microscopy unveiled abnormal mitochondrial cristae ultrastructure in sgCOX4I1‐transduced cells (Figure 3B). Morphometric analysis indicated an increased cristae lumen width (Figure 3C), suggestive of a disorganized mitochondrial inner membrane and cristae junction assembly. We further assessed the status of OPA1,^[^ ^16^ ^]^ a mitochondrial dynamin‐like GTPase crucial for maintaining cristae structure (Figure 3D, left panels). Our findings demonstrated a reduction in membrane‐anchored long‐form OPA1 (L‐OPA1) and an accumulation of soluble short‐form OPA1 (S‐OPA1) upon COX4I1 loss (Figure 3D, right panels), which is associated with the widened cristae structure.^[^ ^17^ ^]^ The opening of cristae junctions in COX4I1‐deficient cells facilitated the release of cytochrome c from mitochondria to the cytosol (Figure 3E), potentially triggering the intrinsic apoptosis pathway. These results underscore the pivotal role of COX4I1 in maintaining mitochondrial homeostasis and anti‐apoptosis mechanisms in leukemia cells.

### 2.3 COX4I1 Controls Mitochondrial Respiration, Energy Metabolism, and Complex IV Assembly in Leukemia

**Figure 4:**
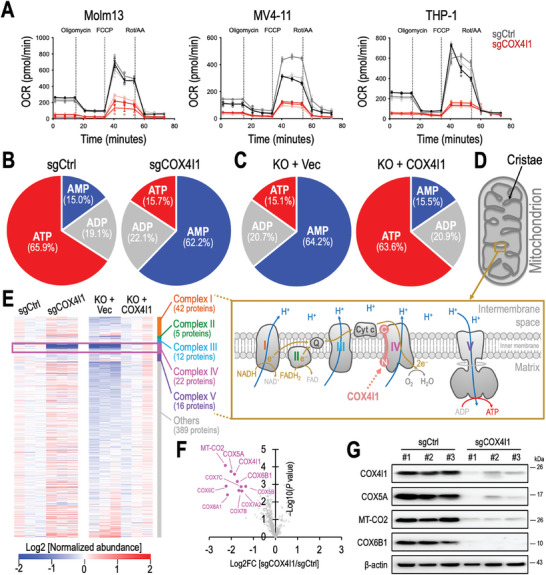
COX4I1 regulates mitochondrial respiration and Complex IV formation. A) Oxygen consumption rate (OCR) measured in Cas9‐expressing Molm13 (left), MV4‐11 (middle), and THP‐1 (right) cells transduced with sgCtrl (gray; *n* = 3 independent sgRNA sequences) and sgCOX4I1 (red; *n* = 3 independent sgRNA sequences). (B and C) Relative levels of AMP, ADP, and ATP in B) Molm13‐Cas9^+^ cells transduced with sgCtrl versus sgCOX4I1, and C) COX4I1‐knockout (KO) Molm13 cells transduced with an empty vector (Vec) versus a synthetic human COX4I1 cDNA. D) Schematic representation of mitochondrial oxidative phosphorylation comprising electron transport chain complexes (I – IV) and ATP synthase (V). H^+^ denotes a proton, and e^–^ represents an electron. Q indicates ubiquinone. E) Heatmaps displaying the relative abundance of the mitochondrial proteome (487 proteins) in (left) Molm13‐Cas9^+^ cells transduced with sgCtrl versus sgCOX4I1, and (right) COX4I1‐KO Molm13 cells transduced with an empty vector versus a synthetic human COX4I1 cDNA. F) Volcano plot comparing mitochondrial protein abundances in Molm13‐Cas9^+^ cells transduced with sgCtrl and sgCOX4I1. Proteins significantly depleted (*P* < 0.01 and Log2FC < −1) in sgCOX4I1 cells are highlighted in pink. G) Western blot analysis of COX4I1, COX5A, MT‐CO2, COX6B1, and β‐actin in Molm13‐Cas9^+^ cells transduced with sgCtrl (*n* = 3 independent sgRNA sequences) and sgCOX4I1 (*n* = 3 independent sgRNA sequences). Data are presented as mean ± SEM.

The impact of sgCOX4I1 on mitochondrial homeostasis prompted us to investigate the role of COX4I1 in mitochondrial respiration, i.e., the process of energy conversion of substrates into ATP. Using the Seahorse XF Cell Mito Stress Test (Agilent) to measure the oxygen consumption rate (OCR), we found that depletion of COX4I1 severely diminished the mitochondrial respiration in all aspects, including the ATP‐linked respiration, maximal respiration, and the reserve respiration capacities in leukemia cells (**Figures** **4**A and S2, Supporting Information). These results highlighted a pronounced defect in mitochondrial function and perhaps a dismissed ATP production in the COX4I1 depeleted cells. To evaluate the role of COX4I1 in cellular energy metabolism, we quantified levels of AMP, ADP, and ATP in Molm13 leukemia cells using mass spectrometry and observed a pronounced reduction of ATP‐to‐AMP ratio upon COX4I1 depletion (Figure 4B). Conversely, the depleted cellular energy state can be readily reversed by ectopic expression of a synthetic human COX4I1 cDNA (Figures 4C and S3, Supporting Information; containing synonymous mutations to produce wild‐type COX4I1 protein without targeting by sgCOX4I1), suggesting the crucial role of COX4I1 protein in mitochondrial integrity and cellular ATP biogenesis.

In mitochondria, the production of ATP is highly regulated by the respiratory electron transport chain (Complex I–IV) and the ATP synthase (Complex V) located on the mitochondrial inner membrane (Figure 4D).^[^ ^18^ ^]^ Complex I receives electrons from nicotinamide adenine dinucleotide (NADH) and passes them to ubiquinone (Q). Q also accepts electrons from flavin adenine dinucleotide (FADH2) and Complex II, passing these electrons to Complex III and cytochrome c (cyt c). COX4I1 is a member of Complex IV (also known as cytochrome c oxidase), the last enzyme in the respiratory electron transport chain. It is critical to receive the donor electrons from cyt c and transfer them to the oxygen molecules as the final electron acceptors. During this process, Complex I, III, and IV transport protons (H^+^) across the inner membrane, increasing the electrochemical potential (i.e., mitochondrial membrane potential or MMP) between the mitochondrial intermembrane space and matrix. This flow of electrons generates a proton gradient across the inner mitochondrial membrane, which is then used by ATP synthase (complex V) to produce ATP from adenosine diphosphate (ADP) in a process called oxidative phosphorylation. Using tandem mass tag (TMT) mass spectrometry, we monitored the level of 487 mitochondrial proteins from Molm13 leukemia cell mitochondrial extracts (Figure 4E; Source Data S5, Supporting Information). Of note, sgCOX4I1 transduction induced a selective impact on Complex IV (Figure 4E, left panel), where 10 out of the 22 Complex IV members (including COX4I1) were significantly depleted (*p* < 0.01 and Log2FC < −1) compared to the sgCtrl transduced cells (Figure 4F, pink dots). Immunoblotting also validated the reduction of the top 4 most significantly depleted mitochondrial proteins (Figure 4G, including COX4I1, COX5A, MT‐CO2, and COX6B1). On the other hand, the depleted Complex IV proteins in the COX4I1 knockout cells can be rescued by ectopic expression of the synthetic human COX4I1 cDNA (Figure 4E, right panel), marking the requirement of COX4I1 in maintaining an intact assembly of Complex IV.

### 2.4 CRISPR Gene Tiling Scan of COX4I1 Identifies Critical Checkpoints Mediating Complex IV Assembly

**Figure 5:**
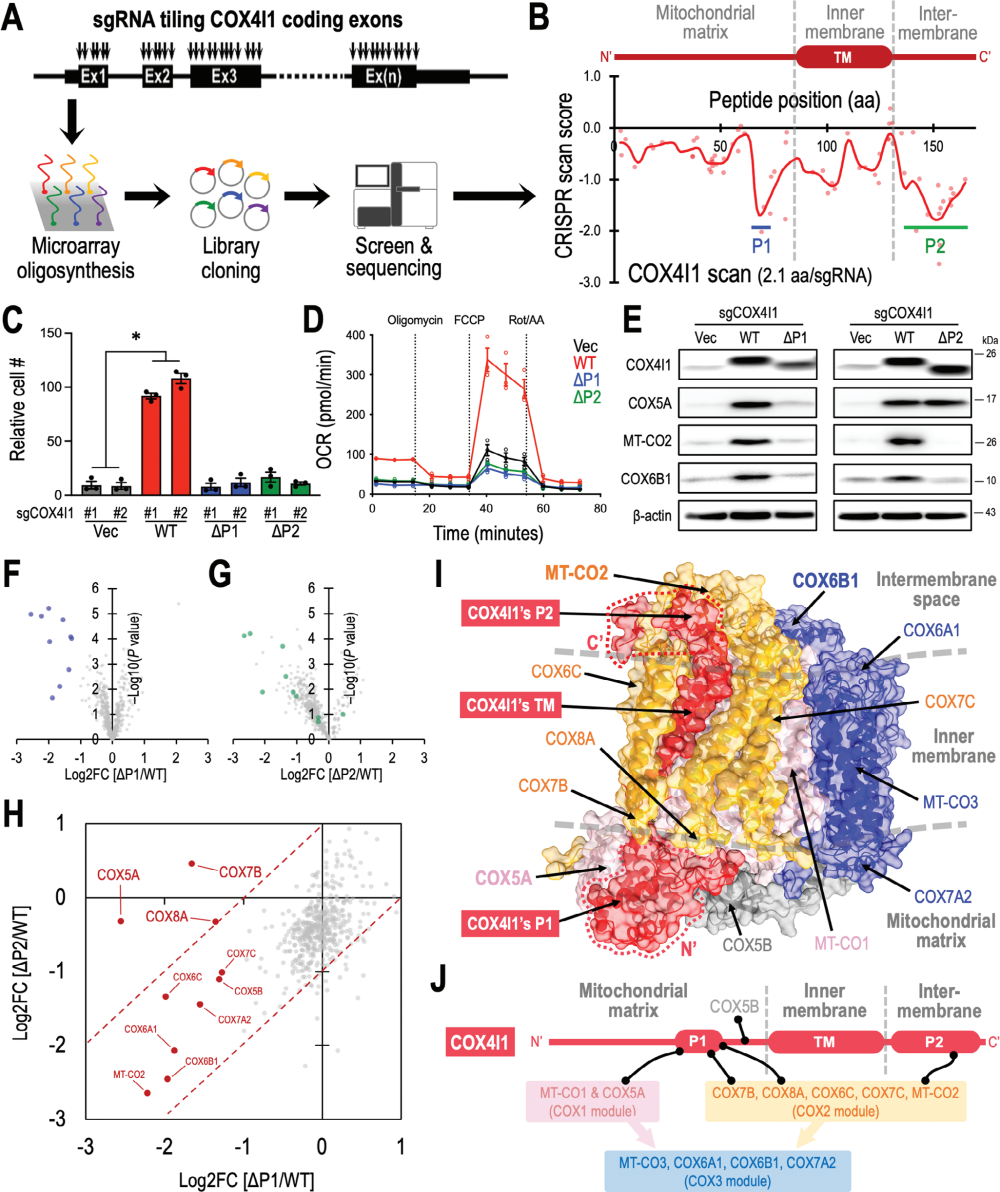
COX4I1 governs pivotal stages of Complex IV assembly. A) Schematic depiction of the COX4I1 high‐resolution CRISPR tiling library screen conducted in Molm13‐Cas9^+^ cells. B) 2D annotation illustrating COX4I1 peptide position (x‐axis) and CRISPR tiling score (y‐axis). The red line represents the smoothed model of the CRISPR scan score derived from individual sgRNAs (dots) targeting the coding exons of COX4I1. The CRISPR hypersensitive P1 and P2 regions are indicated. C,D) Impact of wild‐type (WT), ΔP1, and ΔP2 COX4I1 expression on (C) cell number expansion and (D) mitochondrial respiration in COX4I1‐KO Molm13 cells (*n* = 3 for each group). E) Western blot analysis of COX4I1, COX5A, MT‐CO2, COX6B1, and β‐actin in COX4I1‐KO Molm13 cells transduced with WT, ΔP1, and ΔP2 COX4I1. F,G) Volcano plots comparing mitochondrial protein abundances in COX4I1‐KO Molm13 cells transduced with (F) ΔP1 versus WT COX4I1 and (G) ΔP2 versus WT COX4I1. H) Comparative analysis of the impact of ΔP1 (x‐axis) and ΔP2 (y‐axis) COX4I1 on the mitochondrial proteome (dots). Dashed lines denote a 10‐fold difference between ΔP1 and ΔP2. COX4I1‐regulated mitochondrial proteins defined in Figure 4F are highlighted with (F) blue dots, (G) green dots, and (H) red dots. I) Human Complex IV structural model (PDB 5Z62) colored by COX1 module (pink), COX2 module (orange), and COX3 module (blue). The P1, P2, and transmembrane (TM) regions of COX4I1 are highlighted in red. J) Network model illustrating critical interactions (black lines) between COX4I1 and other components of Complex IV. Data are presented as mean ± SEM. ^*^ *p* < 0.01 by two‐sided Student's *t*‐test.

To delineate the regions essential for leukemia cell survival within COX4I1, we employed a high‐resolution gene tiling scan technique, which identifies functional domains within a protein through CRISPR‐induced mutagenesis.^[^ ^19^ ^]^ To accomplish this, we constructed a CRISPR library targeting every position harboring an NGG protospacer adjacent motif (PAM) within the coding exons of COX4I1 (**Figure** **5**A; SourceData S6 and Figure S1C, Supporting Information). Subsequently, we delivered this library into Cas9‐expressing Molm13 cells using lentiviral transduction and assessed the frequencies of each sgRNA sequence before and after a 21‐day culture through NextSeq550 sequencing (SourceData S7, Supporting Information). Following the refinement of CRISPR results using a local smoothen model,^[^ ^20^ ^]^ our CRISPR tiling scan unveiled the pivotal roles of two regions, P1 (K67–S74 in the matrix domain) and P2 (K135–K169 comprising the intermembrane space domain) within COX4I1, that is critical for leukemia cell survival (Figure 5B).

Functionally, ectopic expression of wild‐type (WT) COX4I1 cDNA, but not the P1‐deleted (ΔP1) or P2‐deleted (ΔP2) variants, rescued impaired cellular survival (Figure 5C) and mitochondrial respiration (Figure 5D) in COX4I1 knockout leukemia cells. Notably, expression of ΔP2 (but not ΔP1) COX4I1 maintained levels of COX5A protein (Figure 5E). We further examined the impact of WT, ΔP1, and ΔP2 COX4I1 on 487 mitochondrial proteins using TMT mass spectrometry (following the same methods as illustrated in Figure 4E,F). Compared to WT expression, ΔP1 exhibited a more pronounced effect on Complex IV protein expression than ΔP2, suggesting distinct regulations of COX4I1's P1 and P2 regions in mitochondrial Complex IV formation (Figure 5F,G; highlighted dots represent the top 10 depleted Complex IV proteins observed in Figure 4F). Cross‐comparison of mass spectrometric data between ΔP1 and ΔP2 revealed the selective requirement of the P1 region of COX4I1 for maintaining COX5A and two other Complex IV members, COX7B and COX8A (Figure 5H).

Structurally, mitochondrial Complex IV can be classified into three primary sub‐complexes.^[^ ^21^ ^]^ MT‐CO1 (pink), COX5 (pink), and COX4I1 (red) proteins are believed to initially assemble into a COX1 module before further incorporating the COX2 (orange; including MT‐CO2, COX6C, COX7B, COX7C, COX8A, etc.) and COX3 (blue; including MT‐CO3, COX6A1, COX6A2, COX7A2, etc.) modules to generate a fully assembled Complex IV (Figure 5I; PDB ID: 5Z62).^[^ ^21a^ ^]^ Our investigation revealed that COX4I1's P1 region closely interacts with COX5A, essential for attaching COX5A (a peripheral membrane protein) to the matrix side of the mitochondrial inner membrane (Figure 5I, bottom‐left and Figure 5J). Moreover, the P1 region contributes to stabilizing the matrix ends of COX7B and COX8A (members of the COX2 module). Conversely, COX4I1's P2 region primarily interacts with MT‐CO2 at the intermembrane space surface of the mitochondrial inner membrane (Figure 5I, top‐left and Figure 5J). The ability of COX4I1 to bridge both the COX1 and COX2 modules represents a critical checkpoint in controlling the formation of Complex IV for mitochondrial respiration (Figure 5J).

### 2.5 Targeting COX4I1 Improves the Efficacy of Venetoclax Treatment in Leukemia Cells

**Figure 6:**
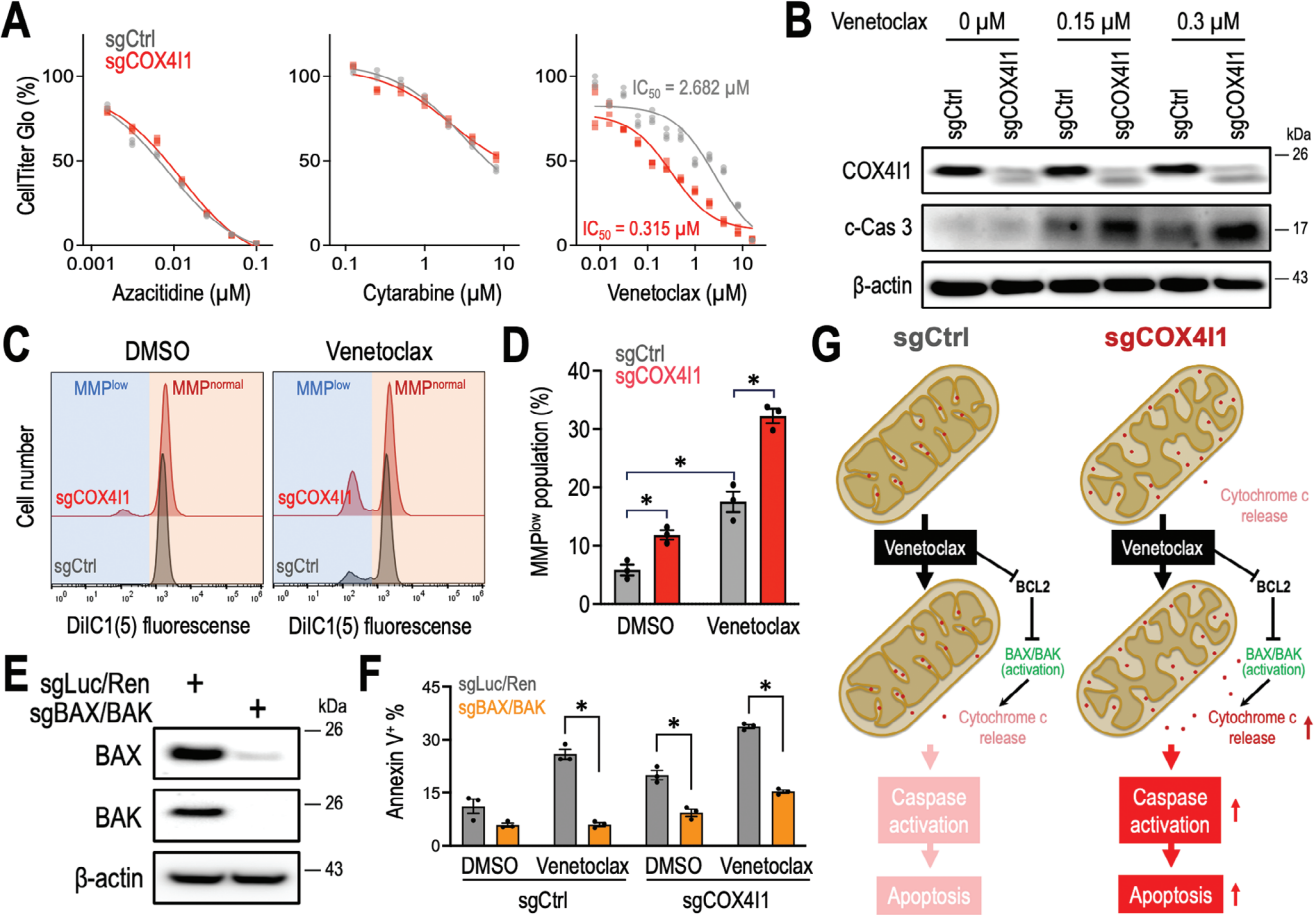
Targeting COX4I1 sensitizes AML cells to venetoclax treatment. A) Impact of targeting COX4I1 on leukemia cells treated with azacytidine, cytarabine, and venetoclax. B) Effect of targeting COX4I1 on protein levels of COX4I1, cleaved caspase 3 (c‐Cas3), and β‐actin in leukemia cells treated with venetoclax. C) Representative flow cytometric profiles of mitochondrial membrane potential (MPP), and D) the effect of targeting COX4I1 on venetoclax‐induced mitochondrial depolarization (*n* = 3 for each group). E) Western blot analysis of BAX, BAK, and β‐actin in leukemia cells transduced with sgLuc/Ren and sgBAX/BAK. F) Effect of targeting COX4I1 on venetoclax‐induced apoptosis in leukemia cells transduced with sgLuc/Ren and sgBAX/BAK (*n* = 3 for each group). G) Schematic model illustrating the enhancement of venetoclax‐induced apoptosis through COX4I1 targeting. Data are presented as mean ± SEM. ^*^ *p* < 0.01 by two‐sided Student's *t*‐test.

The impact of COX4I1 knockout on leukemia cell survival prompted us to explore its potential combinatorial benefits with standard AML regimens such as azacitidine, cytarabine, and venetoclax. We found that sgCOX4I1 transduction did not affect the response of leukemia cells to azacitidine and cytarabine. However, leukemia cells exhibited nearly a 10 fold higher sensitivity to venetoclax (also known as ABT‐199) when COX4I1 was depleted (**Figure** **6**A). Venetoclax is known to induce mitochondrial‐related apoptosis by blocking pro‐survival BCL2 proteins and inducing a caspase‐mediated proteolytic cascade.^[^ ^22^ ^]^ Depletion of COX4I1 augmented the level of cleaved‐caspase 3 (c‐Cas 3; enzymatically active form) in venetoclax‐treated leukemia cells (Figure 6B). Venetoclax also impairs mitochondrial function by shifting leukemia cells from a normal mitochondrial membrane potential (MMP^normal^) to a low mitochondrial membrane potential state (MMP^low^) (Figure 6C,D). We found that CRISPR depletion of COX4I1 depolarized MMP, and the combination of sgCOX4I1 and venetoclax treatment significantly increased the MMP^low^ population within live cells (Figure 6C,D). To evaluate the involvement of mitochondrial apoptosis underlying sgCOX4I1 and venetoclax treatment, we generated a leukemia cell model depleted of the apoptosis effectors BAX and BAK (Figure 6E).^[^ ^23^ ^]^ We observed that cellular apoptosis, as measured by Annexin V positivity (Figure 6F), was efficiently induced by either venetoclax or sgCOX4I1 and further enhanced by their combination in control cells (sgLuc/Ren; sgRNAs targeting *Luciferase* and *Renilla* genes). In contrast, apoptosis induced by these treatments was significantly blocked in sgBAX/BAK transduced cells (Figure 6F), suggesting that the combinatorial effects of venetoclax and COX4I1 knockdown involve the BAX/BAK‐mediated mitochondrial apoptosis pathway. Together with the data observed in Figure 3B,E, we propose that COX4I1 depletion disrupts cristae architecture and primes mitochondrial cytochrome c release, thereby facilitating venetoclax‐induced apoptosis (Figure 6G). Collectively, our study suggests the potential to enhance venetoclax's anti‐leukemia efficacy through combinatorial targeting of COX4I1.

**Figure 7:**
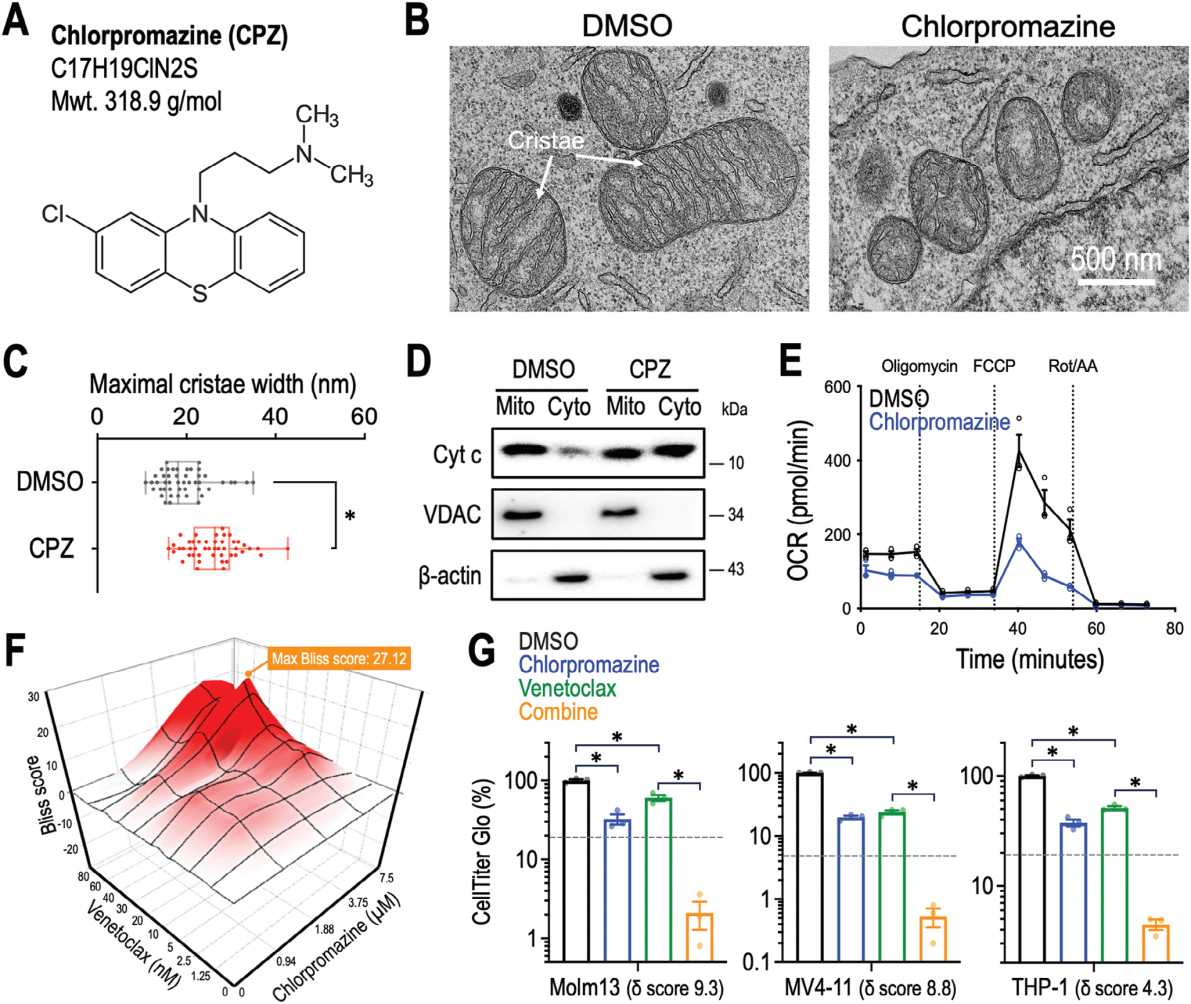
Effect of targeting AML by chlorpromazine. A) Chemical structure of chlorpromazine (CPZ). B) Representative images from transmission electron microscopy depicting mitochondrial ultrastructure and C) measurement of maximal cristae width observed in Molm13 cells incubated with DMSO and chlorpromazine. D) Distribution of cytochrome c (cyt c), voltage‐dependent anion channel (VDAC; a mitochondrial marker protein), and β‐actin (a cytoplasmic marker protein) between the mitochondrial (mito) and cytoplasmic (cyto) fractions of Molm13 cells incubated with DMSO and chlorpromazine. E) Oxygen consumption rate (OCR) measured in Molm13 cells incubated with DMSO and chlorpromazine (*n* = 3 for each condition). F) Bliss synergy test between chlorpromazine and venetoclax in Molm13 cells. G) Effect of DMSO (gray), chlorpromazine (blue), venetoclax (green), or the combination of chlorpromazine and venetoclax (orange) on the survival (measured by CellTiter Glo) and synergy index (δ score) of Molm13 (left), MV4‐11 (middle), and THP‐1 (right) leukemia cells (*n* = 3 for each group). Data are presented as (C) box‐and‐whisker plots illustrating median (center line), first and third quartiles (box), and data range (whiskers), and (E and G) mean ± SEM. ^*^ *p* < 0.01 by two‐sided Student's *t*‐test.

While there is currently no clinical compound that selectively targets COX4I1, we noted that chlorpromazine (CPZ; **Figure** **7**A), an FDA‐approved antipsychotic medication, was reported to suppress chemoresistant COX4I1‐expressing glioma cells by inhibiting mitochondrial Complex IV activity.^[^ ^24^ ^]^ Treatment of chlorpromazine resulted in disorganized mitochondrial cristae ultrastructure (Figure 7B), widened cristae lumen (Figure 7C), facilitated cytochrome c release from mitochondria to the cytosol (Figure 7D), and reduced mitochondrial respiration (Figure 7E) in Molm13 cells. These findings closely parallel the impacts observed in COX4I1 deficient cells (see Figure 3B,C,E, and 4A). As CRISPR targeting COX4I1 increased the sensitivity of AML cells to venetoclax (Figure 6), we further sought to investigate the capacity of chlorpromazine to potentiate the anti‐leukemia effects of venetoclax. Our results revealed that the combination of chlorpromazine and venetoclax exerted a peak Bliss synergy score of 27.12 in Molm13 cells (Figure 7F; Bliss score >10 indicates the synergistic relationship between the two tested compounds). We also observed a synergy index (δ score) ranging between 4.3 and 9.3 in three tested AML cell models (Figure 7G), highlighting the potential use of the chlorpromazine/venetoclax combination for improved leukemia therapy.

## 3 Discussion

Cell signaling pathways encompass a plethora of structural and signaling components that orchestrate various biological activities in both normal and diseased conditions. A deeper comprehension of the cell signaling protein genes associated with cancer progression can unveil novel therapeutic avenues and elucidate previously unknown mechanisms of drug action. In this study, we conducted a series of CRISPR genetic screens in leukemia cells, including a cell signaling pathway‐focused screen and a high‐density COX4I1 CRISPR tiling screen. Through these functional genetic approaches, we uncovered the pivotal role of COX4I1, a mitochondrial protein encoded by the nuclear genome, in leukemia progression. We also illustrated that the CRISPR‐hypersensitive regions within COX4I1 are indispensable for mitochondrial Complex IV formation, unveiling a novel mechanism of nuclear control over mitochondrial function. This discovery potentially provides a new rationale for improving leukemia therapy.

COX4I1, an integral component of mitochondrial Complex IV (cytochrome c oxidase), is pivotal in mitochondrial respiration. Complex IV plays a critical role in the electron transport chain, facilitating the transfer of electrons from cytochrome c to oxygen, ultimately leading to the production of ATP.^[^ ^21a^ ^]^ Mutations or deficiencies in COX4I1 have been associated with growth regression, chromosome instability, intellectual disability, and type 2 diabetes.^[^ ^25^ ^]^ Moreover, COX4I1 expression is dynamically regulated to modulate mitochondrial activity, while elevated levels have been linked to therapy resistance in glioblastoma.^[^ ^24^ ^]^ COX4I1's isoform, COX4I2, though typically expressed at lower levels, may play a more significant role during hypoxic conditions when COX4I1 expression is downregulated.^[^ ^26^ ^]^ Our study underscores that COX4I1 is essential to leukemia cell proliferation and in vivo AML progression (Figure 2). Analysis of the consortium databases highlighted a pronounced expression and gene dependency on COX4I1 in blood cancers (Figure 1C,D). Notably, COX4I2 remained undetectable in RNA‐seq data from COX4I1 knockout AML cells, suggesting a dominant utilization of COX4I1 in AML cells.

The biogenesis of Complex IV involves multiple‐stage coordination of 14 core subunits alongside other chaperone proteins, initially forming three sub‐complexes: COX1, COX2, and COX3 modules.^[^ ^18^, ^21^ ^]^ Given that Complex IV mediates the final step of the electron transport chain, the orchestration of its assembly serves as a pivotal checkpoint for controlling mitochondria energy production throughput. Interestingly, only 3 Complex IV subunits (MT‐CO1, MT‐CO2, and MT‐CO3) were encoded by the mitochondrial DNA, while the majority of the Complex IV members are encoded within the nuclear genome. Various chaperones, including COX15, COX17, COX20, HIGD2A, and SCO1/2, have been implicated in diverse aspects of Complex IV assembly.^[^ ^21^, ^27^ ^]^ While depletion of these chaperones can damage the Complex IV production, none of them showed an elevated expression in the blood cancer patient samples (Figure S4, Supporting Information; GENT2 database). In contrast, our investigation revealed significant overexpression of COX4I1 in blood cancer patients compared to normal blood samples (Figure 1D). Notably, COX4I1 depletion detrimentally affects the protein levels of other Complex IV core subunits (Figure 4E–G) without altering their RNA expression (Figure S5, Supporting Information), suggesting post‐transcriptional regulation and impaired protein complex stability. Further, CRISPR targeting of these COX4I1‐regulated Complex IV members (such as COX5A, COX6B1, etc.) also exhibits a selective impact on blood cancer cells (Figure S6, Supporting Information), emphasizing a unique addiction of leukemia cells to the intact functional Complex IV. Notably, while our study primarily focused on AML cell models with MLL gene rearrangements (Molm13, MV4‐11, and THP‐1), analysis of the DepMap CRISPR screen database indicates that targeting COX4I1 may have broader implications across multiple AML subtypes (Figure S7, Supporting Information), potentially paving the way for therapies against diverse forms of leukemia.

An increasing interest in targeting mitochondrial function was proposed in AML therapy.^[^ ^28^ ^]^ For example, it has been reported that disruption of mitochondrial ultrastructure could sensitize AML to venetoclax, a standard‐of‐care medication assigned for chronic lymphocytic leukemia (CLL) and AML patients.^[^ ^15^, ^22^ ^]^ Our study revealed that genetic targeting COX4I1 facilitates the cellular apoptosis induced by venetoclax (Figure 6A–F), which could be attributed to the distorted mitochondrial architecture in the COX4I1 deficient AML cells (Figure 3B). The loosen cristae junction (i.e., widen cristae with reduced L‐OPA1 to S‐OPA1 ratio shown in Figure 3C,D) and the leakiness of mitochondria (increased cytochrome c release into cytosol observed in Figure 3E) induced by sgCOX4I1 preconditioned the AML cells as amendable to venetoclax (a BH3 mimetic) treatment, which primes cytochrome c release and the caspase‐mediated apoptosis through inhibiting the pro‐survival BCL2 proteins and activation of the pro‐apoptotic BAX/BAK proteins (Figure 6G).^[^ ^2^, ^22^ ^]^

Chlorpromazine is a medication for managing symptoms of psychotic disorders such as hallucinations, delusions, and disorganized thinking. It acts primarily as a dopamine receptor antagonist in the brain.^[^ ^29^ ^]^ Recently, chlorpromazine has been investigated for its potential therapeutic effects in other conditions. For example, it has been studied for its antiemetic properties and potential to enhance chemotherapy's effects in cancer treatment.^[^ ^24^ ^]^ The anti‐AML capacity of chlorpromazine was also recently identified.^[^ ^30^ ^]^ Interestingly, chlorpromazine was known to inhibit the mitochondrial Complex IV activity, providing a pharmacological tool that mimics the blocking of COX4I1 in leukemia cells. We observed that similar to CRISPR inhibition of COX4I1, chlorpromazine treatment potentiates the efficacy of venetoclax in suppressing leukemia cell survival (Figure 7F,G). Of note, the synergistic effect between chlorpromazine and venetoclax was also detected in THP‐1, a venetoclax‐resistant AML cell line.^[^ ^31^ ^]^ These observations indicate a possibility of repurposing chlorpromazine, an FDA‐approved medication, to improve the outcome of AML patients experiencing venetoclax resistance.

While our study primarily focused on the role of mitochondrial respiration and apoptotic mechanisms, the significant disruption of mitochondrial architecture associated with COX4I1 depletion may have broader implications. For instance, we hypothesized that defective mitochondria in COX4I1‐deficient cells might induce mitophagy, a selective degradation process of mitochondria via autophagy.^[^ ^32^ ^]^ However, we did not observe an increased number of autophagosomes or autolysosomes engulfing mitochondria in COX4I1‐depleted cells (Figure S8, Supporting Information), suggesting that mitophagy is not a significant contributor to the cellular stress induced by COX4I1 depletion. On the other hand, mitochondria play a central role in fatty acid oxidation, iron metabolism, and ROS production. Our transcriptomic analysis revealed that COX4I1 deficiency also triggers ferroptosis (Figure S9A, Supporting Information), a form of iron‐dependent cell death marked by increased lipid peroxidation (Figure S9B, Supporting Information) and diminished glutathione‐dependent antioxidant defenses (Figure S9C, Supporting Information).^[^ ^33^ ^]^ Inhibition of ferroptosis using liproxstatin‐1 (Lip‐1)^[^ ^34^ ^]^ resulted in ≈20% rescue of cells in the sgCOX4I1‐transduced population (Figure S9D, Supporting Information), indicating that ferroptosis partially contributes to cell death following COX4I1 depletion.

High‐throughput CRISPR library screens across various cancer cell types have illuminated crucial mechanisms governing tumorigenesis and therapeutic responses.^[^ ^10^, ^35^ ^]^ For instance, genome‐wide CRISPR screens have identified essential mitochondrial components necessary for diverse nutritional sources.^[^ ^36^ ^]^ Conversely, the potential of CRISPR technology to elucidate gene function at a sub‐gene level, such as protein domains or motifs, is being actively explored.^[^ ^37^ ^]^ High‐resolution CRISPR gene tiling screens have delineated essential elements within functional domains and have demonstrated sensitivity in identifying therapeutic pockets and drug interaction sites within screened proteins.^[^ ^12^, ^38^ ^]^ Here, we incorporated the high‐resolution CRISPR tiling of COX4I1 with mitochondrial proteomics together with 3D structural analysis and pinpointed the roles of P1 (K67–S74) and P2 (K135–K169) regions and their interacting networks as critical checkpoints mediating Complex IV assembly (Figure 4). We foresee this multi‐omics approach will be highly applicable to other research aiming at dissecting the protein domain functions and therapeutic target discovery.

## 4 Experimental Section

### Cell Lines and Culture Conditions

Cell lines including Molm13, MV4‐11, THP‐1, and HEK293 were procured from the American Type Culture Collection (ATCC). Molm13, MV4‐11, and THP‐1 cells were maintained in Roswell Park Memorial Institute (RPMI) 1640 Medium (Thermo Fisher Scientific), supplemented with 10% fetal bovine serum (FBS; Omega Scientific). HEK293 cells were cultured in Dulbecco's Modified Eagle Medium (DMEM; Gibco), supplemented with 10% FBS. All media formulations were supplemented with penicillin (100 units mL^−1^; Gibco), streptomycin (100 µg mL^−1^; Gibco), l‐alanyl‐l‐glutamine dipeptide (2mM; GlutaMAX, Gibco), and plasmocin (0.5 µg mL^−1^; InvivoGen). Cultures were maintained in a humidified incubator at 37 °C with 5% CO_2_. Cells expressing the Cas9 endonuclease were generated by transduction with LentiCas9‐Blast (52962, Addgene) lentivirus, followed by selection with blasticidin (Gibco).

### CRISPR and cDNA Molecular Cloning

Guide RNA oligonucleotides were synthesized either via microarray (CustomArray) for library cloning or individually (Integrated DNA Technologies) for single sgRNA constructs. These oligonucleotides were then cloned into the ipUSEPR lentiviral sgRNA vector, which contains hU6‐driven sgRNA co‐expressed with EF‐1α‐driven red fluorescent protein (RFP) and a puromycin‐resistance gene, using BsmBI restriction sites (Figure S1A, Supporting Information). The cell signaling pathway CRISPR library encompassed 3416 sgRNA sequences targeting 427 human genes, with an allocation of 8 sgRNAs per gene (SourceData S2 and Figure S1B, Supporting Information), was designed using the BROAD Institute Genetic Perturbation Platform–CRISPick.^[^ ^35c^ ^]^ For the COX4I1 gene tiling scan CRISPR library, 82 sgRNA sequences covering every protospacer adjacent motif (PAM) within the human COX4I1 coding exons were designed, resulting in an average coverage of 6.2 base pairs per sgRNA (SourceData S6 and Figure S1C, Supporting Information). The integrity of the cloned libraries was assessed through NextSeq sequencing, ensuring that at least 90% of the sgRNA sequences exhibited a minimal read count of ten reads per million reads (RPMR). Individual sgRNAs targeting non‐essential sequences (sgCtrl; 5′‐GATTCTAAAACGGATTACCA‐3′, 5′‐GGATGATAACTGGTCCGCAG‐3′, and 5′‐GAAGATGGGCGGGAGTCTTC‐3′) and COX4I1 coding regions (sgCOX4I1; 5′‐GAACTTAATGCGATACACTG‐3′, 5′‐GGGTGGCCAAGCAGACCAAG‐3′, and 5′‐ATGGGCAGCTGGGCCAGGGC‐3′) were selected for validation experiments. For COX4I1 expression, a full‐length COX4I1 cDNA containing synonymous mutations to evade targeting by sgCOX4I1 was designed utilizing the CLC Main Workbench software and synthesized via gBlock Gene Fragments (Integrated DNA Technologies). Subsequently, the synthetic COX4I1 cDNA was cloned into the ECEG lentiviral vector (features EF‐1α‐driven transgene co‐expression with green fluorescent protein) by Gibson Assembly (New England Biolabs). The final plasmids were confirmed via Sanger sequencing (Eton Bioscience).

### Lentiviral Production and Transduction

Lentiviruses were produced using HEK293 cells (CRL‐1573, ATCC) along with the packaging plasmids pPAX2 (12260, Addgene) and pMD2.G (12259, Addgene). The plasmids pPAX2, pMD2.G, and a lentiviral backbone plasmid (ipUSEPR‐sgRNA or ECEG‐cDNA) were combined in a 1:1:1 ratio in Opti‐MEM medium (31‐985‐062, Gibco) supplemented with 50 µg mL^−1^ polyethyleneimine (PRIME‐P100‐100MG, Serochem LLC). 24 h post‐transfection of HEK293 cells, the culture medium was replaced with fresh DMEM medium, and cells were allowed to incubate for an additional 48 h to facilitate lentivirus production. Subsequently, the supernatants containing lentiviral particles were subjected to precipitation by overnight incubation with 10% polyethylene glycol (BP233‐1, ThermoFisher Scientific) at 4 °C, followed by centrifugation at 3000 g, 4 °C for 30 min to collect the viral pellets. The precipitated viral particles were resuspended in an appropriate RPMI medium, aliquoted, and stored at −80 °C until further use. For lentiviral transduction, target cells were exposed to the viral solution supplemented with polybrene (8 µg mL^−1^; MilliporeSigma) and subjected to centrifugation at 1000 rpm, 37 °C for 60 min.

### CRISPR Library Screens

The lentivirus containing CRISPR libraries was pre‐titrated to achieve ≈15% infection, as assessed by flow cytometry for RFP expression, in Molm13 cells stably expressing the Cas9 endonuclease. For the cell signaling pathway library screen, 35 million cells were used per replicate, while 2 million cells were used for the COX4I1 CRISPR tiling scan. Following infection with the designated CRISPR library, cells were selected with puromycin (1 µg mL^−1^; Gibco). Subsequently, the library‐transduced cells were sub‐cultured every four days. Genomic DNA was collected at the initiation (day 0) and conclusion (day 28 for the cell signaling pathway screen and day 21 for the COX4I1 tiling screen) of the screens. Integrated guide RNA was PCR‐amplified using DCF01 5′‐CTTGTGGAAAGGACGAAACACCG‐3′ and DCR03 5′‐CCTAGGAACAGCGGTTTAAAAAAGC‐3′ primers and subjected to high‐throughput sequencing. The frequency of individual guide RNAs was calculated by mapping 20‐nucleotide sequences matching the guide RNA backbone structure to the library guide RNA sequences. The top essential candidate genes for the cell signaling pathway screen were analyzed using the Model‐based Analysis of Genome‐wide CRISPR‐Cas9 Knockout (MAGeCK) algorithm.^[^ ^9^ ^]^ For the COX4I1 CRISPR gene tiling scan, the CRISPR scan score was defined as the log10 fold change in sgRNA frequency between start and end points, normalized by control sgRNAs. Under‐represented sgRNAs (less than 5% of average frequency) were excluded from the analysis. The CRISPR tiling score was further interpolated by Gaussian kernel smoothing in R, and the average score over the trinucleotide codons was calculated for each peptide position.^[^ ^7b^ ^]^

### Flow Cytometry and Biochemical Assays

In competition cell culture assays, Cas9‐expressing cells were transduced with ipUSEPR (RFP^+^) sgRNA constructs in 96‐well plates, aiming for ≈50% infection efficiency. The relative RFP% was utilized to quantify the proportion of RFP^+^ cells at different time points post‐lentiviral infection compared to the RFP^+^% observed on day 0. Cell viability was assessed through the exclusion of 4′,6‐diamidino‐2‐phenylindole (DAPI; D1306, Invitrogen) dye for live cells. Cytofluorometric monitoring of mitochondrial membrane potential (MMP) was executed using the MitoProbe DiIC1(5) assay kit (M34151, Thermofisher). Data were obtained by high‐throughput flow cytometry using an Attune NxT flow cytometer with an autosampler (Thermo Fisher Scientific). Lipid peroxidation levels were assessed using a malondialdehyde (MDA) assay kit (ab233471, Abcam), whereas the relative glutathione (GSH) and glutathione disulfide (GSSG) ratio were calculated using a GSH/GSSG ratio detection kit (ab138881, Abcam).

### Western Blotting

Cell lysates were prepared by incubating 5 million cells per ml in LDS sample buffer (Invitrogen) at 95 °C for 10 min. Electrophoretic separation of the samples was carried out using Bolt 4–12% Bis‐Tris plus gels (NW04125, Invitrogen), followed by transfer onto PVDF membranes (0.2 µm pore size; IB24002, Invitrogen) using PVDF Mini Stacks and iBlot 2 (Invitrogen). PVDF membranes were subsequently blocked with 5% bovine serum albumin (Fisher Scientific) in Tris‐buffer saline‐tween 20 (TBST) at room temperature for 1 h. Primary antibodies targeting COX4I1 (rabbit mAb [clone 3E11]; 4850, Cell Signaling Technology, 1:1000), OPA1 (rabbit mAb [clone D6U6N]; 80471, Cell Signaling Technology, 1:1000), cytochrome c (rabbit mAb [clone EPR1327]; ab133504, Abcam, 1:1000), VDAC (rabbit mAb [clone D73D12]; 4661, Cell Signaling Technology, 1:1000), COX5A (rabbit mAb [clone EPR14208(B)]; ab180129, Abcam, 1:1000), MT‐CO2 (rabbit mAb [clone E6U9K]; 50003, Cell Signaling Technology, 1:1000), COX6B1 (mouse mAb [clone 3F9D3D11AF6]; ab110266, Abcam, 1:500), cleaved caspase 3 (rabbit mAb [clone 5A1E]; 9664, Cell Signaling Technology, 1:1000), BAX (rabbit mAb [clone D2E11], 5023, Cell Signaling Technology, 1:1000), BAK (rabbit mAb [clone D4E4], 12105, Cell Signaling Technology, 1:1000), and β‐actin (mouse mAb [clone 8H10D10]; 3700, Cell Signaling Technology; 1:1000) were then applied to the membranes at 4 °C overnight. Following washing steps, the membranes were incubated with HRP‐conjugated goat anti‐mouse (31430, Invitrogen; 1:200000) or goat anti‐rabbit (31460, Invitrogen; 1:200000) IgG antibodies at room temperature for 1 h. Chemiluminescent signals were developed using the SuperSignal West Femto Substrate (P134095, ThermoFisher) and detected using a ChemiDoc imaging system (Bio‐Rad).

### In Vivo AML Modeling

The “human‐in‐mouse” xenograft leukemia model was established by transplanting 6–8 weeks old NSGS mice (strain ID: NOD.Cg‐Prkdc^scid^ Il2rg^1Wjl^ Tg(CMV‐IL3, CSF2, KITLG)1Eav/MloySzJ; RRID: IMSR_JAX:013062; The Jackson Laboratory) with human leukemia cells.^[^ ^12b^ ^]^ NSGS mice were assigned randomly to the experimental groups. Molm13‐Cas9^+^ human AML cells underwent transduction with pLenti CMV Puro LUC (17477, Addgene) lentiviruses, followed by selection with 2 µg mL^−1^ puromycin for 4 days to generate luciferase‐expressing cells conducive to bioluminescence imaging. Subsequently, these Molm13‐Cas9^+^/Luc^+^ cells were transduced with ipUSEPR lentiviruses expressing sgCtrl and sgCOX4I1. The sgRNA expressing cells were isolated via RFP^+^ fluorescence‐activated cell sorting. To establish the in vivo AML model, 0.2 million sorted cells were suspended in PBS and transplanted into NSGS mice via tail vein injection. Leukemia progression was monitored through weekly in vivo bioluminescence imaging of the recipient mice. D‐luciferin (4, 5‐Dihydro‐2‐(6‐hydroxy‐2‐benzothiazolyl)‐4‐thiazolecarboxylic acid potassium salt; LUCK‐2G, GoldBio) dissolved in PBS was administered via intraperitoneal injection at a dose of 150mg kg^−1^, following a 10 min pre‐imaging interval, and anesthesia with isoflurane. Whole‐body bioluminescence imaging was detected using a Lago × Imager (Spectral Instruments Imaging), with the bioluminescence signal presented in radiance in units of “photons/seconds/cm2/steradian”. Pseudocolors were utilized to denote the strength of the leukemia burden signal. The mice were maintained under a 12 h:12 h light‐dark cycle with ad libitum access to food and water. Euthanasia of the recipient mice was conducted via CO2 inhalation upon the manifestation of systemic illness signs. All animal experiments adhered to institutional guidelines and were conducted in accordance with an IACUC protocol #17098 approved by the City of Hope.

### Mitochondrial Ultrastructural Analysis

Transmission electron microscopy analysis of sgCtrl and sgCOX4I1 transduced Molm13‐Cas9^+^ cells was conducted following established protocols.^[^ ^12a^ ^]^ First, cells were fixed in 2.5% glutaraldehyde in 0.1 m sodium cacodylate buffer (Na(CH3)2AsO2·3H2O), pH 7.2, at 4 °C. Subsequently, samples underwent post‐fixation with osmium tetroxide, followed by serial dehydration using ethanol and embedding in Eponate. Ultra‐thin sections with a thickness of ≈70 nm were obtained via ultramicrotomy, then post‐stained and examined using an FEI Tecnai 12 transmission electron microscope equipped with a Gatan OneView CMOS camera at the City of Hope Electron Microscopy Core Facility.

### Transcriptomic Analysis

For RNA‐seq analysis, total RNA was extracted utilizing the RNeasy Mini Kit (74104, QIAGEN) and subsequently processed for mRNA library preparation. Sequencing was performed on a NovaSeq 6000 platform (paired‐end 150 bp; ≈20 million reads per sample) at Novogene Inc. Raw sequence reads underwent trimming to eliminate poly(A) tails and adapters using Trimmomatics v0.39. Alignment to the Human Genome Assembly GRCh38.p14 was carried out using Bowtie2 v2.5.1, allowing for one mismatch to accommodate genetic polymorphism. RSEM v1.3.3 was employed to estimate read counts and transcripts per million (TPM), a normalized unit for each gene and isoform.^[^ ^39^ ^]^ Differentially‐expressed genes (DEGs) were identified using EdgeR^[^ ^40^ ^]^ and DESeq2,^[^ ^41^ ^]^ with criteria set at an absolute fold change of ≥2 and a false discovery rate (FDR) adjusted p‐value of <0.05. To uncover mechanistically relevant pathways or processes, Gene set enrichment analysis (GSEA) was conducted using GSEA v4.1.0 software.^[^ ^14^ ^]^

### Mitochondrial Respiration Assays

Oxygen consumption rate (OCR) was measured using the Seahorse XF Cell Mito Stress Test kit and a Seahorse XFe96 Extracellular Flux Analyzer (Agilent Technologies). In brief, 1 50 000 cells per well were seeded in XF96 Cell Culture Microplates coated with Cell‐Tak (Corning). Following cell plating, the cells were incubated in XF RPMI medium (without phenol red), supplemented with 2 mM glutamine, 10 mM glucose, 1 mM pyruvate, and 5 mM HEPES for 45 min at 37 °C in a non‐CO2 incubator before the assay initiation. The assay consisted of oxygen consumption and extracellular acidification measurements starting with the basal conditions, followed by sequential injections of Oligomycin 1 µM, FCCP 2.2 µM, and Rotenone/Antimycin 0.5 µM. Three measurements were recorded after each compound injection. The analysis was conducted simultaneously in the same plate for both sgCtrl and sgCOX4I1 transduced cells.

### Mass Spectrometric Analyses

For measuring adenosine phosphate nucleotides (AMP, ADP, and ATP), one million cells were washed with ice‐cold 5% mannitol, and 1 mL cold 80% methanol (−80 °C) was added to cell pellets. Five nmol norvaline (Sigma–Aldrich) was added to each sample as an internal standard. Samples were then vortexed three times over 15 min on ice and spun down at top speed for 5 min. The supernatant was transferred to a new 1.5 mL tube, and the samples were dried down on Vacufuge Plus (Eppendorf) at 30 °C. The mass spectrometric analysis of extracted samples was conducted at UCLA Metabolomics Center. For detecting mitochondrial proteome, mitochondrial proteins from 10 million cells were extracted using the Mitochondria Isolation Kit for Cultured Cells (89874, ThermoFisher) and quantified by DC Protein Assay Kit II (5000112, Bio‐Rad). Mitochondrial proteins were digested using the EasyPepTM Mini MS Sample Prep Kit (Thermo Fisher Scientific, USA). After protein digestion, 10µg of peptides were labelled using the 0.1 mg of TMTpro 18‐plex Label Reagent Set (Thermo Fisher Scientific, USA). The TMTpro labeled samples were pooled and proceeded to peptide clean‐up according to the manufacturer's protocol. Eluted peptides were suspended in 2% acetonitrile (ACN) and 0.2% formic acid (FA) for further LC‐MS/MS analysis. The tandem mass tag mass spectrometry analysis of the mitochondrial protein extracts was performed by the Proteome Exploration Laboratory at Caltech.

### Chlorpromazine and Venetoclax Combination Test

Leukemia cells were seeded at a density of 10 000 cells per well in 96‐well plates and incubated with varying concentrations of chlorpromazine (0–15 µM) and venetoclax (0–100 nM) in combinations for 48 h (*n* = 3 wells per condition). For the cell viability assay, cells were washed twice with phosphate‐buffered saline (PBS) and then resuspended by trypsinization. Subsequently, the resuspended cells (50 µl) were mixed with CellTiter‐Glo 2.0 reagent (10 µl; G9241, Promega) in white flat‐bottom 96‐well plates (353296, Corning) and incubated at room temperature for 10 min. The luminescence emitted was quantified using an Infinite M1000 Pro plate reader (Tecan Trading AG, Switzerland). Relative CellTiter Glo (%) signals were normalized to the control condition (DMSO). The drug combination Bliss synergy score was evaluated using SynergyFinder v3.0.^[^ ^42^ ^]^ The synergy index (δ score) of the chlorpromazine and venetoclax combination was defined as follows:

(1) $$\delta \;\mathrm{score}=\frac{V\%\left(cpz\right)\;x\;V\%\;\left(ven\right)}{V\%\left(cpz+ven\right)\;x\;V\%\left(dmso\right)\;}$$

where *V%(dmso), V%(cpz), V%(ven)*, and *V%(cpz + ven)* were the observed cell viability under DMSO, chlorpromazine alone, venetoclax alone, and chlorpromazine plus venetoclax combination, respectively.

### Statistical Analysis

The sample size (n) for each experiment is indicated in the figure legend. Data were represented as mean ± SEM (for experiments with n ≤ 10) or median ± interquartile range (for experiments with n > 10). Using two tailed unpaired *t*‐test, the differences between the two groups were analyzed. ^*^ *p* < 0.01 was considered as statistically significant. Statistics were performed by GraphPad Prism 9.

### Availability of Materials

Cas9‐expressing cells, COX4I1 cDNA, CRISPR library for cell signaling pathways, and COX4I1 gene tiling scan available upon request. All other biological materials were commercially available.

## Conflict of Interest

Jianjun Chen is a scientific advisory board member of Race Oncology.

## Author Contributions

L.Z., H.Z., T.‐Y.W., M.L., A.K.N.C, Q.L., S.P.P., N.M., P.S., Z.E., B.K. X.W. performed the experiments; A.N.K.C., H.K., L.C.F., L.Y. analyzed the data; S.T.R., J.C., T.‐F.C., R.S. provided conceptual input; L.Z., C.‐W.C. wrote the paper; C.‐W.C. conceived and supervised the study.

## Supporting information



Supporting Information

## Data Availability

The RNA‐seq transcriptomics and mass spectrometry proteomics data generated in this study are available via Gene Expression Omnibus (GEO) under accession GSE261970 and ProteomeXchange Consortium (via the PRIDE) under PXD050739. 3D protein structure of mitochondrial Complex IV (PDB ID: 5Z62) was obtained from the Research Collaboratory for Structural Bioinformatics Protein Data Bank (RCSB PDB). Additional data that support the findings of this study are provided in the Supplementary Information.
