# Supplementary material for: Nuclear Control of Mitochondrial Homeostasis and Venetoclax Efficacy in AML via COX4I1
Source: Adv Sci (Weinh). 2024 Dec 23;12(6):2404620. doi: 10.1002/advs.202404620 (PMC11809339; doi:10.1002/advs.202404620)
Supplement: Supplementary file 1 — Supporting Information [file ADVS-12-2404620-s001.pdf]

ADVANCED SCIENCE
Open Access

Supporting Information

for *Adv. Sci.*, DOI 10.1002/advs.202404620

Nuclear Control of Mitochondrial Homeostasis and Venetoclax Efficacy in AML via COX4I1

*Leisi Zhang, Honghai Zhang, Ting-Yu Wang, Mingli Li, Anthony K.N. Chan, Hyunjun Kang, Lai C. Foong, Qiao Liu, Sheela Pangeni Pokharel, Nicole M. Mattson, Priyanka Singh, Zeinab Elsayed, Benjamin Kuang, Xueer Wang, Steven T. Rosen, Jianjun Chen, Lu Yang, Tsui-Fen Chou, Rui Su and Chun-Wei David Chen**

Supplementary Materials

Nuclear Control of Mitochondrial Homeostasis and Venetoclax Efficacy in AML via COX4I1

Authors:

Leisi Zhang[1,2], Honghai Zhang[1], Ting-Yu Wang[3], Mingli Li[1], Anthony K.N. Chan[1,4], Hyunjun Kang[5], Lai C. Foong[3], Qiao Liu[1], Sheela Pangeni Pokharel[1,4], Nicole M. Mattson[1], Priyanka Singh[1], Zeinab Elsayed[1], Benjamin Kuang[1], Xueer Wang[1], Steven T. Rosen[6], Jianjun Chen[1,6], Lu Yang[1,4], Tsui-Fen Chou[3], Rui Su[1,6], Chun-Wei David Chen[1,4,6,*]

Affiliations:

[1]Department of Systems Biology, Beckman Research Institute, City of Hope, Duarte, CA, USA

[2]National Clinical Research Center for Hematologic Diseases, Jiangsu Institute of Hematology, The First Affiliated Hospital of Soochow University, Suzhou, Jiangsu, China

[3]Proteome Exploration Laboratory, California Institute of Technology, Pasadena, CA, USA

[4]Division of Epigenetic and Transcriptional Engineering, Beckman Research Institute, City of Hope, Duarte, CA, USA

[5]Department of Hematologic Malignancies Translational Science, Beckman Research Institute, City of Hope, Duarte, CA

[6]City of Hope Comprehensive Cancer Center, Duarte, CA, USA

*Corresponding author. Email: cweichen@coh.org.

[*]**Correspondence:**

Chun-Wei (David) Chen, Ph.D.

Associate Professor, Department of Systems Biology

Director, Division of Epigenetics and Transcriptional Engineering

Beckman Research Institute, City of Hope National Medical Center

1500 E. Duarte Rd, Duarte, CA 91010, USA

E-mail: cweichen@coh.org

ORCID: 0000-0002-8737-6830

Fig S1

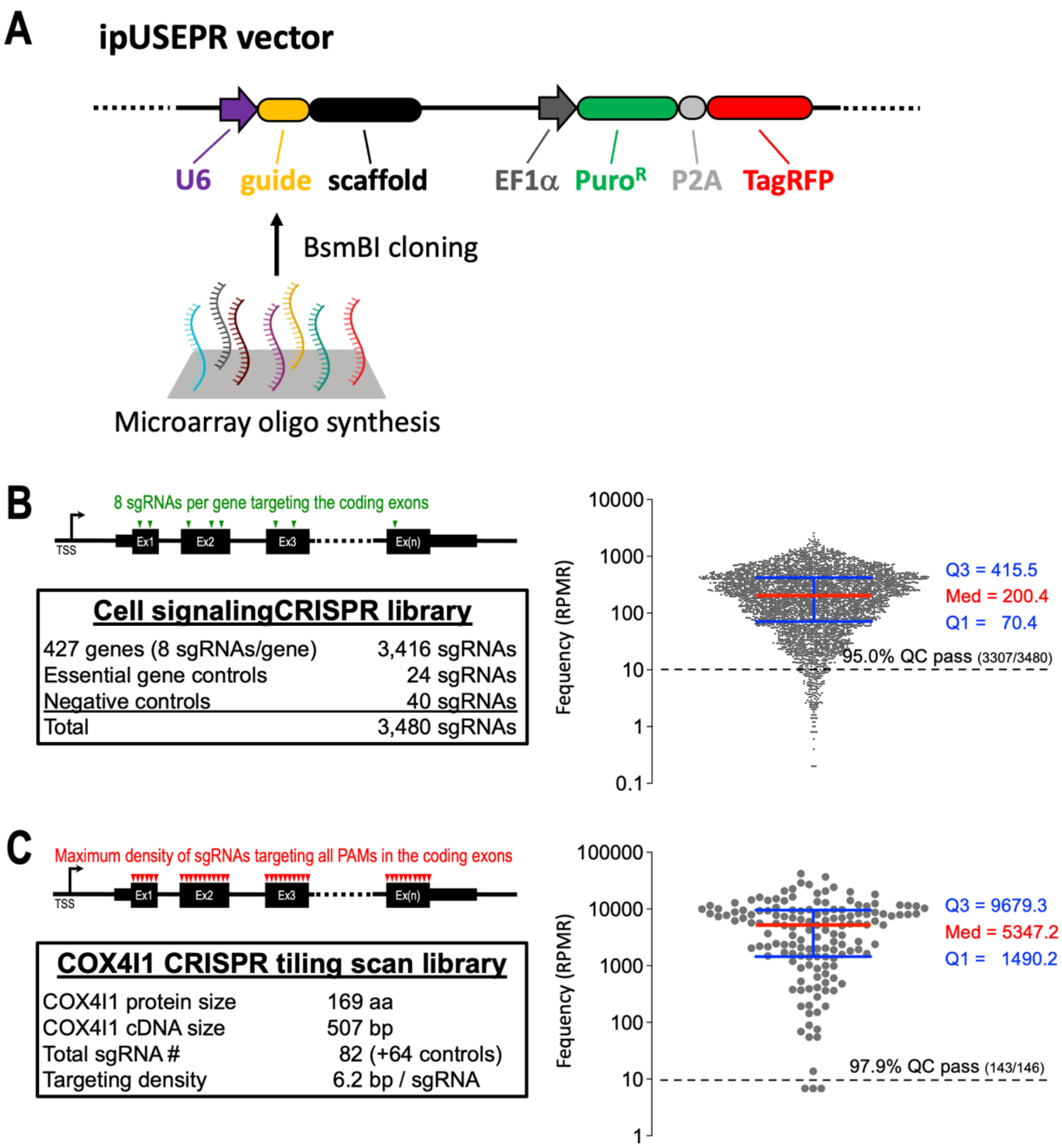

Figure S1. Custom built CRISPR screen libraries used in this study.

(A) Map of the ipUSEPR vector expressing an sgRNA together with a puromycin-resistant gene (PuroR) and a TagRFP fluorescent protein. (B and C) Design and distribution of individual sgRNA frequencies, RPMR (reads per million reads), in the CRISPR libraries targeting (B) cell signaling genes (n = 3,416 sgRNAs) and (C) coding regions of COX4I1 (n = 82 sgRNAs). (B) 95.0% and (C) 97.9% of sgRNAs in these libraries passed QC by exhibiting RPMR $\geqslant$ 10. Data are represented as median $\pm$ interquartile range.

Fig S2

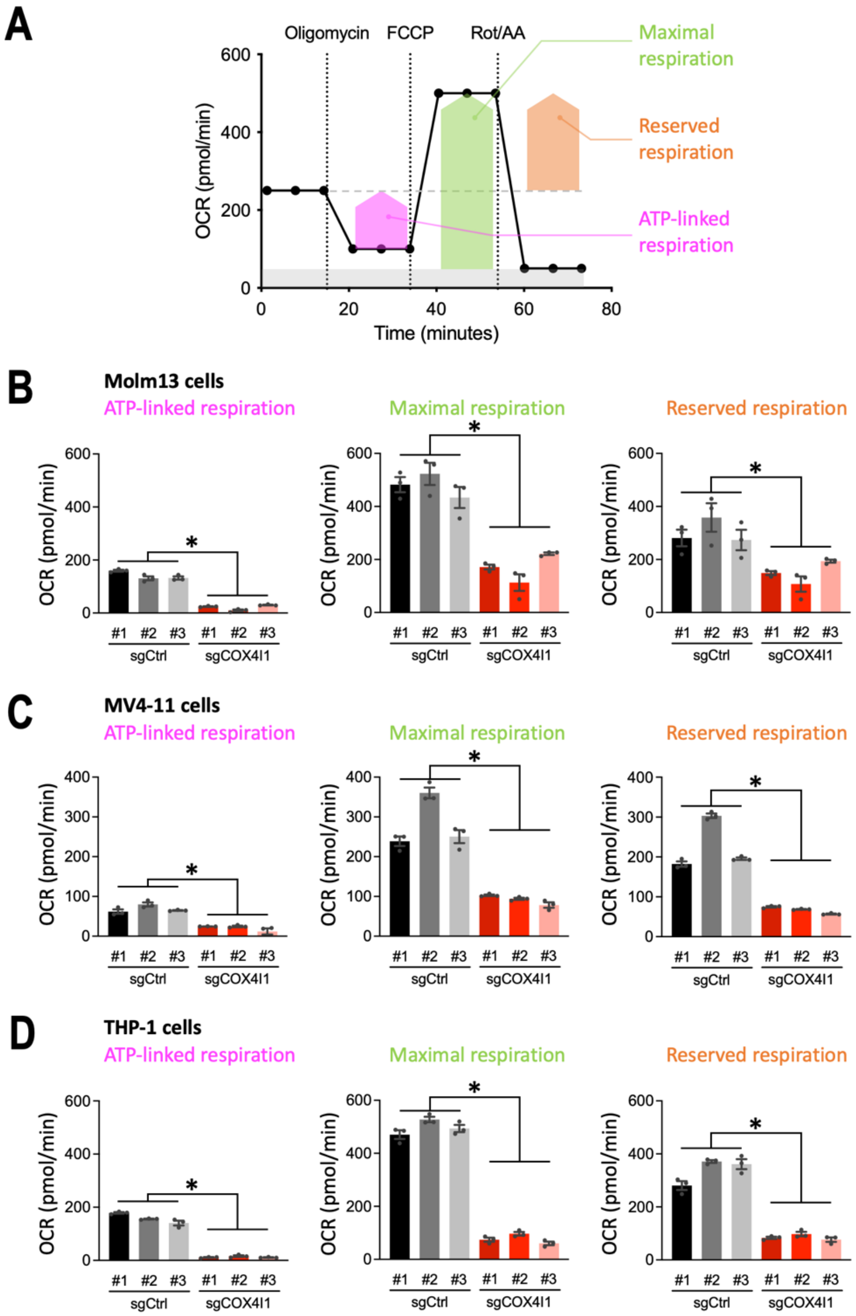

Figure S2. Effect of COX4I1 depletion on mitochondrial respiration in AML cells.

(A) Schematic representation of the Seahorse assay. (B – D) Measurement of ATP-linked respiration (left), maximal respiration (middle), and reserved respiration (right) in Cas9-expressing Molm13 (left), MV4-11 (middle), and THP-1 (right) cells transduced with sgCtrl (gray; n = 3 independent sgRNA sequences) and sgCOX4I1 (red; n = 3 independent sgRNA sequences). Data are presented as mean ± SEM. *P < 0.01 by two-sided Student's t-test

Fig S3

A

<u>**cDNA sequence identies: 390/506 (77.1%)**</u>

```
Endogenous COX4I1    1  ATGTTGGCTACCAGGGTATTTAGCCTAGTTGGCAAGCGAGCAATTTCCAC   50
 Synthetic COX4I1    1  ...C.C..C..AC.T..T..C...T.G..A..A..A..C..C........   50

Endogenous COX4I1   51  CTCTGTGTGTGTACGAGCTCATGAAAGTGTTGTGAAGAGCGAAGACTTTT  100
 Synthetic COX4I1   51  TAGC..C........C..C..C..GTCC..A..C.....T..G.......  100

Endogenous COX4I1  101  CGCTCCCAGCTTATATGGATCGGCGTGACCACCCCTTGCCGGAGGTGGCC  150
 Synthetic COX4I1  101  .C..G..C.....C........TA.G........GC.A..A.....C..G  150

Endogenous COX4I1  151  CATGTCAAGCACCTGTCTGCCAGCCAGAAGGCATTGAAGGAGAAGGAGAA  200
 Synthetic COX4I1  151  .....G.....T..T..G..G..T........GC.A..A...........  200

Endogenous COX4I1  201  GGCCTCCTGGAGCAGCCTCTCCATGGATGAGAAAGTCGAGTTGTATCGCA  250
 Synthetic COX4I1  201  ....AGT...TCT..TT.G..A..............T..A..A.....T.  250

Endogenous COX4I1  251  TTAAGTTCAAGGAGAGCTTTGCTGAGATGAACAGGGGCTCGAACGAGTGG  300
 Synthetic COX4I1  251  .C.....T..A...........C..A..............C.....A...  300

Endogenous COX4I1  301  AAGACGGTTGTGGGCGGTGCCATGTTCTTCATCGGTTTCACCGCGCTCGT  350
 Synthetic COX4I1  301  ..A..A..A.....A..C..A.....T.....T..C.....A..C..A..  350

Endogenous COX4I1  351  TATCATGTGGCAGAAGCACTATGTGTACGGCCCCCTCCCGCAAAGCTTTG  400
 Synthetic COX4I1  351  ...A........A...........A..T..G...T.A..A.....T..C.  400

Endogenous COX4I1  401  ACAAAGAGTGGGTGGCCAAGCAGACCAAGAGGATGCTGGACATGAAGGTG  450
 Synthetic COX4I1  401  .T..G........A..A........A..AC.C..................  450

Endogenous COX4I1  451  AACCCCATCCAGGGCTTAGCCTCCAAGTGGGACTACGAAAAGAACGAGTG  500
 Synthetic COX4I1  451  ..T.....T..A...C....G..T..A...........G..A..T..A..  500

Endogenous COX4I1  501  GAAGAA  506
 Synthetic COX4I1  501  ......  506
```

B

<u>**Peptide sequence identies: 169/169 (100%)**</u>

```
Endogenous COX4I1    1  MLATRVFSLVGKRAISTSVCVRAHESVVKSEDFSLPAYMDRRDHPLPEVAHVKHLSASQK   60
                        MLATRVFSLVGKRAISTSVCVRAHESVVKSEDFSLPAYMDRRDHPLPEVAHVKHLSASQK
 Synthetic COX4I1    1  MLATRVFSLVGKRAISTSVCVRAHESVVKSEDFSLPAYMDRRDHPLPEVAHVKHLSASQK   60

Endogenous COX4I1   61  ALKEKEKASWSSLSMDEKVELYRIKFKESFAEMNRGSNEWKTVVGGAMFFIGFTALVIMW  120
                        ALKEKEKASWSSLSMDEKVELYRIKFKESFAEMNRGSNEWKTVVGGAMFFIGFTALVIMW
 Synthetic COX4I1   61  ALKEKEKASWSSLSMDEKVELYRIKFKESFAEMNRGSNEWKTVVGGAMFFIGFTALVIMW  120

Endogenous COX4I1  121  QKHYVYGPLPQSFDKEWVAKQTKRMLDMKVNPIQGLASKWDYEKNEWKK  169
                        QKHYVYGPLPQSFDKEWVAKQTKRMLDMKVNPIQGLASKWDYEKNEWKK
 Synthetic COX4I1  121  QKHYVYGPLPQSFDKEWVAKQTKRMLDMKVNPIQGLASKWDYEKNEWKK  169
```

Figure S3. Design of the synthetic COX4I1 cDNA construct.

(A) The cDNA sequence of the synthetic COX4I1 construct (containing synonymous mutations) shares 77.1% identity with the endogenous human COX4I1 cDNA. The switched nucleotides in the synthetic COX4I1 cDNA are labeled in red, which facilitates protein expression by optimizing codon usage (CLC Genomics Workbench, QIAGEN). The synthetic COX4I1 cDNA also introduces mismatched sequences within the sgCOX4I1 landing sites, thus allowing reconstitution of COX4I1 protein expression in sgCOX4I1-targeted cells. (B) The peptide sequence of the synthetic COX4I1 construct shares 100% identity with the endogenous human COX4I1 peptide.

Fig S4

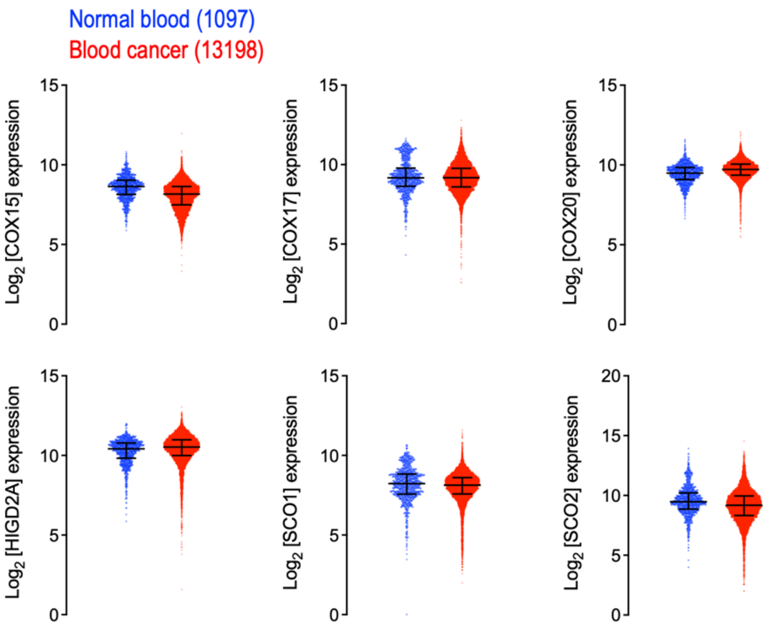

Figure S4. Expression of Complex IV chaperones in normal and blood cancer samples.
Expression levels of COX15, COX17, COX20, HIGD2A, SCO1, and SCO2 in normal and blood cancer samples were obtained from the GENT2 database (http://gent2.appex.kr/gent2/), which collected gene expression data from over 14,000 normal and cancer blood samples.

Fig S5

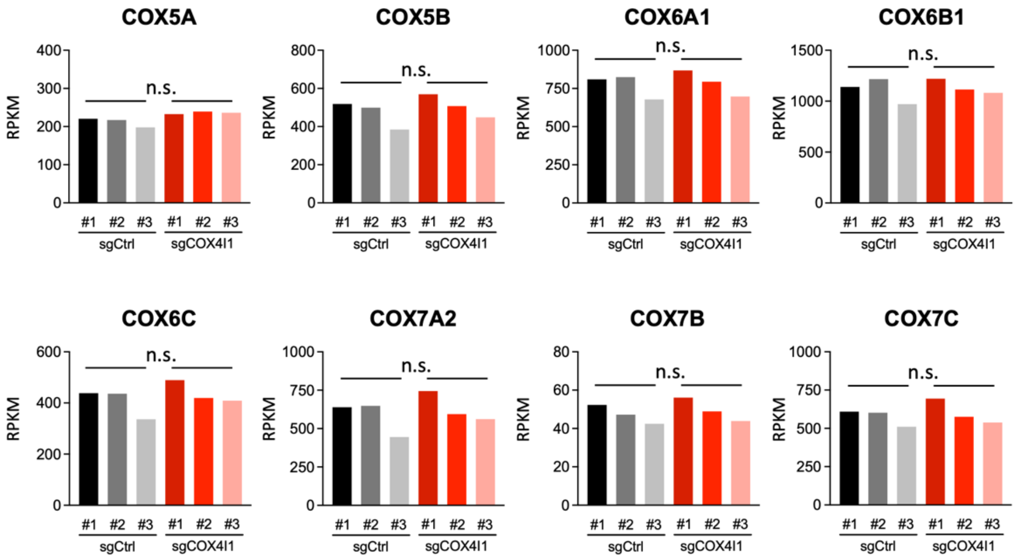

Figure S5. Effect of COX4I1 depletion on the mRNA level of Complex IV members.

Expression levels of COX5A, COX5B, COX6A1, COX6B1, COX6C, COX7A2, COX7B, and COX7C were measured by RNA-seq of Molm13-Cas9^{+} cells transduced with sgCtrl (gray; n = 3 independent sgRNA sequences) and sgCOX4I1 (red; n = 3 independent sgRNA sequences). Data are presented as mean ± SEM. n.s.: not significant by two-sided Student’s t-test.

Fig S6

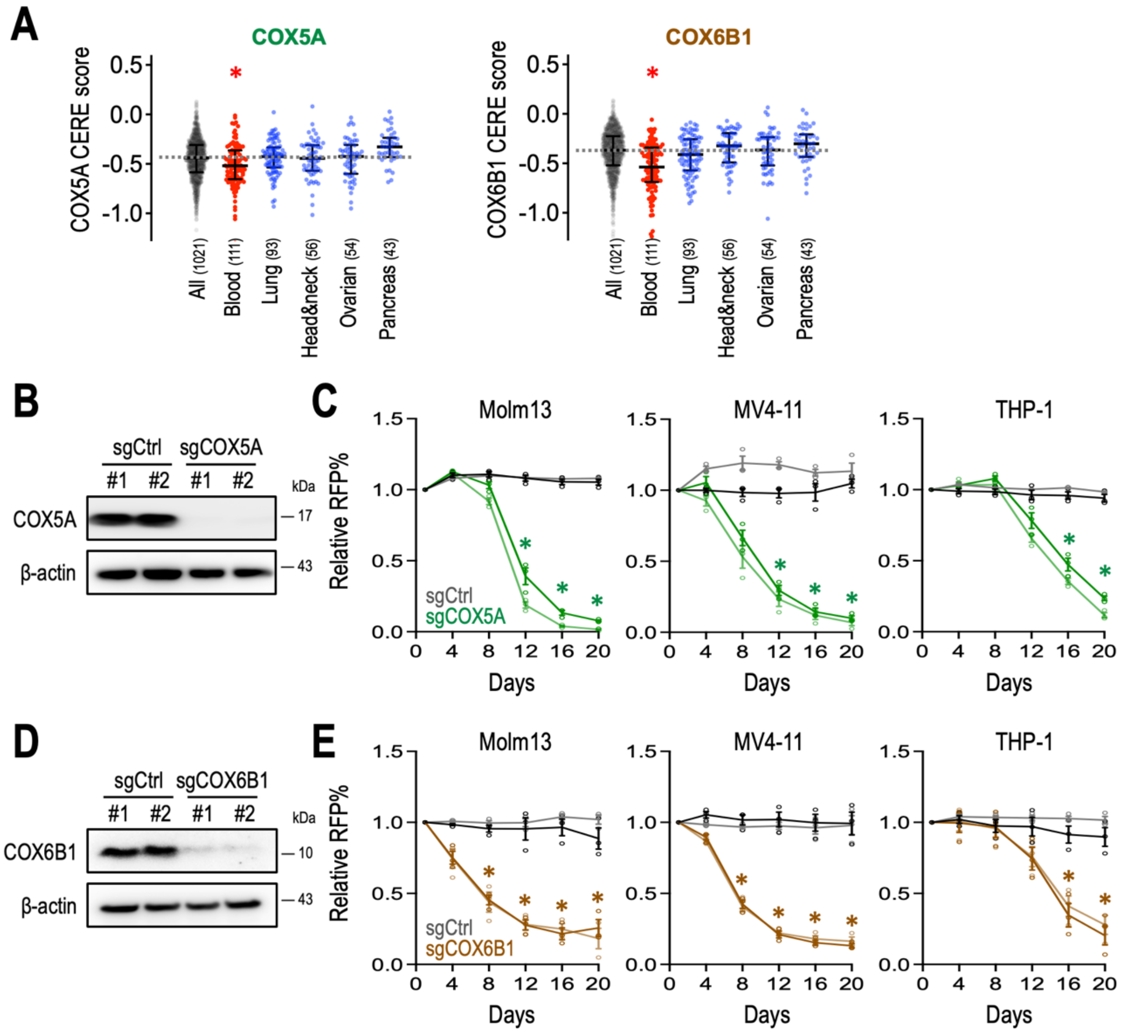

Figure S6. Cancer cell dependency to COX5A and COX6B1.

(A) Dot plots show the CRISPR gene dependency (CERE) score of COX5A (left) and COX6B1 (right) across 1,021 human cancer cell lines tested in the DepMap consortium (BROAD Institute). The selective dependency of blood cancer cells on COX5A and COX6B1 is similar to their dependency on COX4I1, as shown in Fig. 1C. (B) Western blot analysis of COX5A and β-actin in Molm13-Cas9^{+} cells transduced with sgCtrl and sgCOX5A. (C) Growth competition assay of Cas9-expressing Molm13 (left), MV4-11 (middle), and THP-1 (right) cells transduced with RFP-labeled sgCtrl (gray lines; n = 2 independent sgRNA sequences) and sgCOX5A (green lines; n = 2 independent sgRNA sequences). (D) Western blot analysis of COX6B1 and β-actin in Molm13-Cas9^{+} cells transduced with sgCtrl and sgCOX6B1. (C) Growth competition assay of Cas9-expressing Molm13 (left), MV4-11 (middle), and THP-1 (right) cells transduced with RFP-labeled sgCtrl (gray lines; n = 2 independent sgRNA sequences) and sgCOX6B1 (brown lines; n = 2 independent sgRNA sequences). Data are presented as (A) median ± interquartile range and (C and E) mean ± SEM. *P < 0.01 by two-sided Student's t-test.

Fig S7

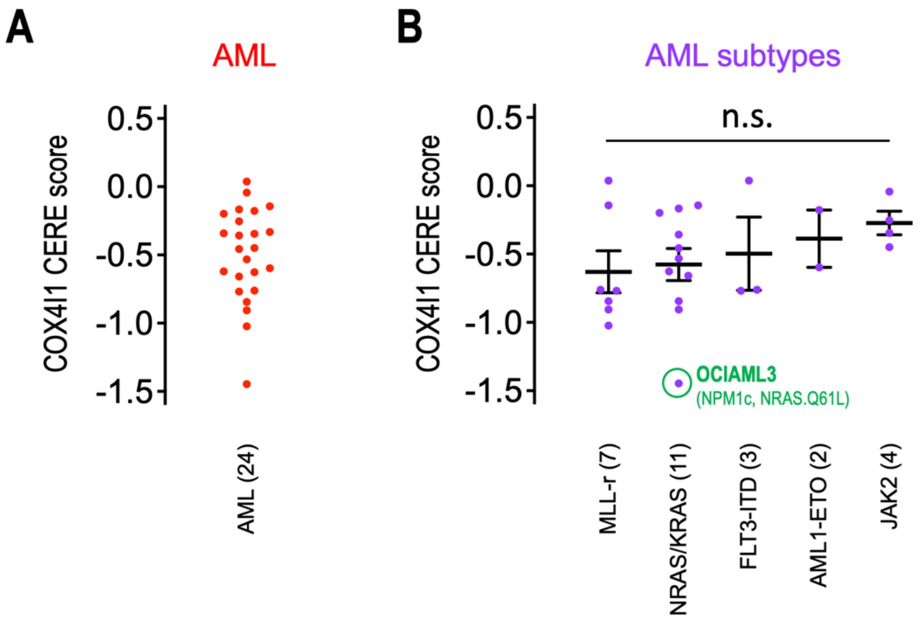

Figure S7. Impact of COX4I1 targeting across various AML cell lines tested in the DepMap consortium. Dot plots showing the CRISPR gene dependency (CERE) score of COX4I1 across (A) all 24 human AML cell lines and (B) several common AML subtypes characterized by abnormalities in MLL-rearrangement (MLL-r), NRAS/KRAS, FLT3-ITD, AML-ETO, and JAK2. MLL-r AML generally exhibited heightened dependency on COX4I1 (more negative CERE score) compared to other subtypes, although these differences did not reach statistical significance. Notably, OCIAML3, an AML cell line harbors mutations in both NPM1 and NRAS (green), showed the highest sensitivity to COX4I1 depletion among all AML cell models tested by the DepMap consortium. Data are presented as mean ± SEM.

Fig S8

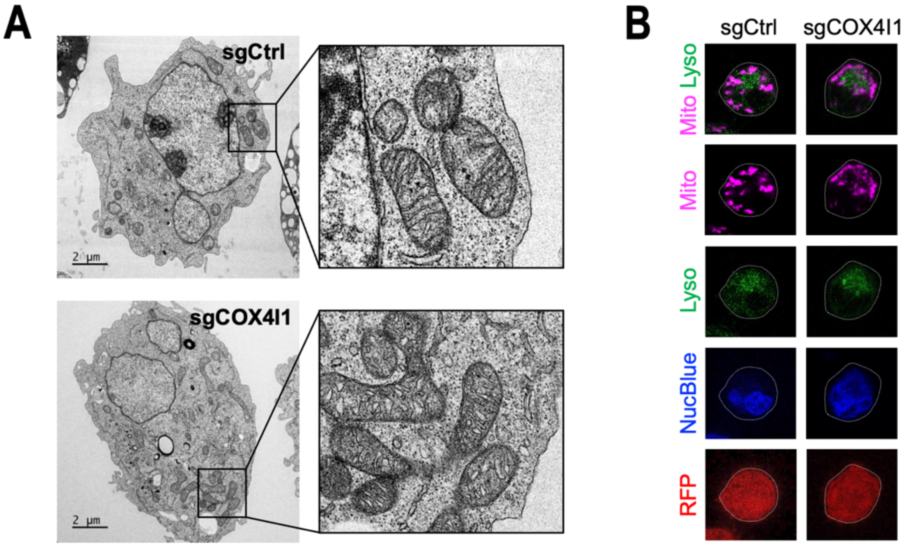

Figure S8. Mitophagy is not a significant factor in sgCOX4I1-induced cellular stress.

(A) Representative images from transmission electron microscopy revealed a pronounced disorganization of mitochondrial ultrastructure in sgCOX4I1-transduced Molm13-Cas9^{+} cells. However, we did not observe an increased number of autophagosomes or autolysosomes in these COX4I1-depleted cells. (B) Confocal microscopy was also used to assess the localization of mitochondria and lysosomes in AML cells. These fluorescent images revealed distinct distributions of mitochondria (pink; MitoTracker™ Deep Red FM, ThermoFisher M22426) and lysosomes (green; lysoSensor green DND-189, ThermoFisher L7535), with minimal colocalization of the two organelles (i.e., autolysosome engulfing mitochondria) in either sgCtrl or sgCOX4I1-transduced Molm13-Cas9^{+} cells. Cell nuclei were stained by NucBlue Hoechst 33342 (ThermoFisher R37605). Red fluorescent protein (RFP) indicates cells transduced with the sgRNA lentivirus.

Fig S9

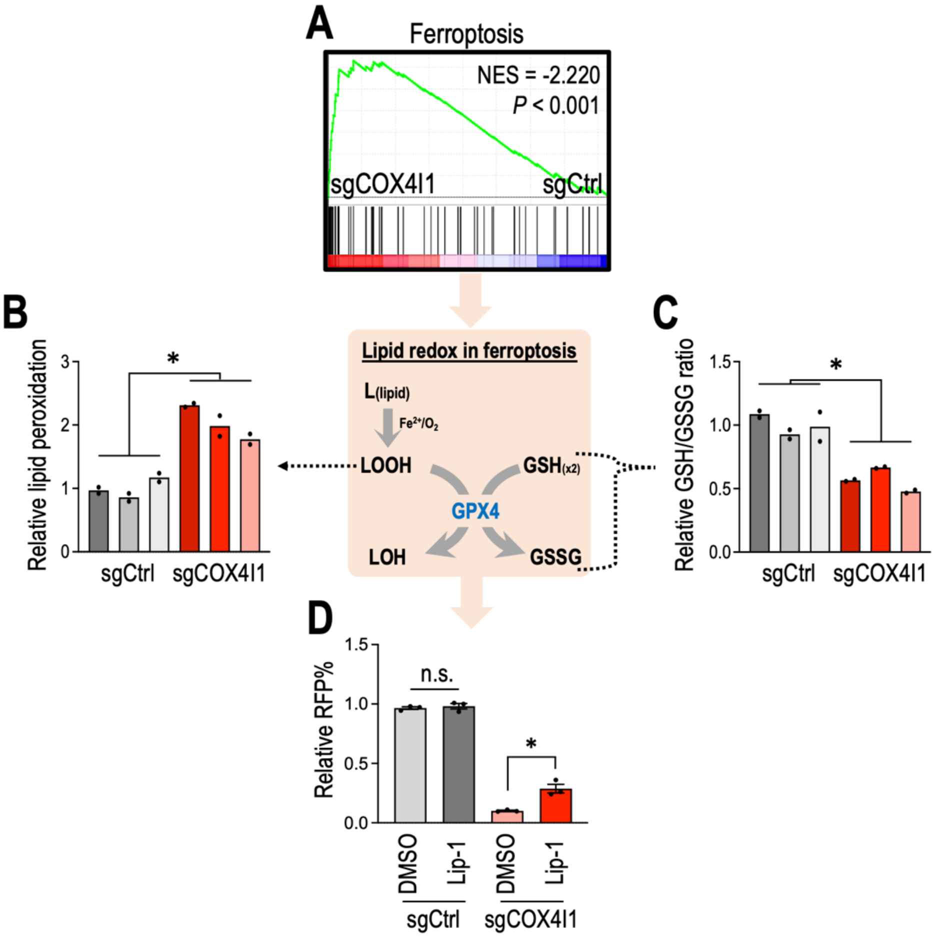

Figure S9. Involvement of ferroptosis in sgCOX4I1-induced cellular stress.

(A) RNA-seq and GSEA analyses indicating an upregulation of ferroptosis-related genes in sgCOX4I1-transduced Molm13-Cas9+ cells. (B) Measurement of lipid peroxidation and (C) analysis of GSH/GSSG ratio in Molm13-Cas9+ cells transduced with sgCtrl (gray; n = 3 independent sgRNA sequences) and sgCOX4I1 (red; n = 3 independent sgRNA sequences). (D) Inhibition of ferroptosis by liproxstatin-1 (Lip-1) partially rescued sgCOX4I1 transduced Molm13-Cas9+ cells. LOOH: lipid hydroperoxides. LOH: lipid alcohols. GPX4: glutathione peroxidase 4. Data are presented as mean ± SEM. *P < 0.01 by two-sided Student's t-test.
